# Liquid fertilizer recovered from the effluent water of recirculating aquaculture system by reverse osmosis exhibits storage stability independent of pasteurization

**DOI:** 10.1371/journal.pone.0355602

**Published:** 2026-08-21

**Authors:** Didrik Ulleberg, Gema Raspati, Tara Baele, Mari Birkeland, Jon Brage Svenning

**Affiliations:** 1 SINTEF Nord, Tromsø, Norway; 2 Group of Water and Environment, SINTEF Community, Trondheim, Norway; 3 Kytos, Zwijnaarde, Belgium; 4 Columbi Farms, Stokksundveien, Refsnes, Norway; Indian Institute of Technology Delhi, INDIA

## Abstract

The shift to a circular bioeconomy is dependent on green-tech innovations that upcycle waste streams into reusable components. The current study explores the chemical and microbiological stability of a fertilizer concentrate produced from effluent water of recirculating aquaculture systems (RAS) using reverse osmosis. The objective was to assess the shelf life of the concentrate and determine whether pasteurization is required to prevent spoilage during storage in light-proof containers at room temperature for six months. The chemical and microbiological storage stability of the pasteurized concentrate was monitored by periodic sampling and compared to an untreated control. Pasteurization decreased the initial concentration of elements dissolved in the concentrate, the viable cell load and the percentage of intact bacterial cells. However, both the pasteurized and the untreated control maintained chemical and microbiological stability during storage, indicating that the concentrate has a shelf life of at least 6 months independent of pasteurization. The concentrate is therefore suitable for long-term storage, without the increased production costs and energy use required for pasteurization, thereby providing a reliable and circular source of plant nutrients that have the potential to support sustainable farming practices. This study will contribute to the continued transition towards sustainable aquaculture by repurposing RAS effluent as a valuable by-product for crop cultivation.

## 1. Introduction

Modern agriculture is dependent on mineral fertilizers to produce sufficient food for the still-growing world population. Norway is no exception; according to Statistics Norway, a total of 87,000 tons of nitrogen and 7,000 tons of phosphorus were utilized from mineral fertilizers in 2023 [1]. The production of nitrogen fertilizer requires hydrogen from natural gas and is highly energy-intensive. It accounts for about 2% of global energy consumption and 450 Mt of CO_2_ emissions annually [2]. Meanwhile, phosphorus mineral fertilizer production relies on declining phosphate rock reserves, most of which are located in Morocco and Western Sahara [3]. As a result, fertilizer prices and availability are increasingly volatile due to energy market instability and rising geopolitical tensions.

At the same time, Norwegian aquaculture releases vast quantities of organic and inorganic nutrients into the ocean as waste. A 2021 study estimated that Norwegian mariculture released 60,000 tons of nitrogen and 14,000 tons of phosphorus annually [4]. Most of these nutrient salts are released directly into the ocean through open net-pen production and are therefore unavailable for nutrient recovery, with the exception of locations with infrastructure for sludge collection [5]. However, increased production of Atlantic salmon (*Salmo salar*) and rainbow trout (*Oncorhynchus mykiss*) in recirculating aquaculture systems (RAS) could enable nutrient recovery from water and sludge waste streams [6]. In 2020, land-based aquaculture facilities in Norway were estimated to release 925 tons of nitrogen and 149 tons of phosphate annually [4].

Recirculating aquaculture systems are land-based fish cultivation facilities characterized by a high degree of water reuse [7]. To maintain optimal water quality in the rearing tanks, water is circulated through several treatment steps such as mechanical filtration, oxygenation, CO_2_ degassing, pH adjustment, sludge removal, and biofilters. Within the biofilter, ammonia excreted by the fish is converted to nitrite and then to nitrate by nitrifying bacteria, resulting in nitrate accumulation over time. Therefore, some water exchange is necessary to prevent nutrient salts from accumulating to toxic levels. Effluent water is sometimes treated with biological denitrification to reduce eutrophication before discharge [8], and solids and sludge are removed by filtration.

To increase the sustainability of land-based aquaculture, nutrient salts accumulated in RAS effluent water should ideally be recovered and used as plant fertilizer. However, there are currently no technologies implemented in commercial scale facilities that enable nutrient recovery. Thus, the transition to a circular bioeconomy will require novel solutions that enable nutrient recovery from waste streams, thereby alleviating the consumption of mineral fertilizer.

Although nitrate accumulates in the water of RAS during recirculation, its final concentration remains low compared to that of commercial liquid fertilizers [9]. The water could be used directly for fertilizer in aquaponic cultivation systems, but in most cases the nutrient concentration is too low to support optimal plant growth without supplementation with mineral fertilizer [10]. Furthermore, the amount of mineral salts generated in a full-scale RAS in Norway exceeds the fertilizer requirements of local farms, necessitating transport to other locations. Consequently, widespread use of RAS effluent water as a plant fertilizer depends on water-treatment technologies, such as reverse osmosis (RO), that concentrate nutrient salts. Production of a concentrate allows for efficient transportation, storage, and distribution, while simultaneously producing clean water that can increase the degree of water recirculation in RAS.

Finally, the commercial viability of any organic fertilizer depends on its storage stability, as these products are typically acquired in bulk and utilized gradually over the duration of a growth season. Untreated organic fertilizers may be susceptible to spoilage during storage due to their high water activity and organic load. They may degrade through microbiological growth of algae, fungi, and bacteria, or by chemical changes such as precipitation, oxidation, and enzymatic reactions [11]. Controlling storage conditions such as ambient temperature, pH, oxygen availability, and light exposure is therefore essential to preserve nutrient availability, and to stabilize the microbiological activity in the product. Conventional preservative methods such as pasteurization may also be necessary to improve the storage stability by inhibiting the growth of microorganisms [12].

In the current study, a RO system was used to recover plant nutrients from salmon RAS effluent water. While alternative technologies like nanofiltration and forward osmosis can fulfil the same function, RO was chosen due to its maturity and extensive application in existing water treatment infrastructure. The aim of the study was to monitor the storage stability of the resulting RO concentrate over six months, with and without pasteurization. This study represents the first investigation of the long-term storage stability of an inorganic nutrient concentrate suitable for use as a nitrogen-rich fertilizer produced from RAS effluent water. By demonstrating how underutilized organic waste streams can be converted into storage-stable nutrient concentrates, the study promotes holistic approaches to food production and can contribute to reducing the use of mineral fertilizers in crop production.

## 2. Materials and methods

### 2.1. Production of nutrient concentrate from RAS effluent water

Field activities were conducted at the NIBIO Landvik research station (Norwegian Institute of Bioeconomy Research, Norway). Access to the site and approval for experimental activities were granted by research station manager Randi Seljåsen. RAS effluent water was sourced from a research-scale RAS facility at the site. Salmon (*Salmo salar)* were reared in 1,000 L tanks at a density between 70–100 kg/m^3^ and fed with Nutra RC® feed (Skretting, Stavanger, Norway)*.* The RAS system included two fish rearing tanks*,* a moving bed biofilter to convert ammonia to nitrate, and a hydrocyclone in combination with a drum filter for removal of feces and particles. The oxygen level was kept at 100% saturation, with the use of pure oxygen in nanobubbles, while CO_2_ was removed from the water by a CO_2_ scrubber. The pH was kept at 6.85 by buffering with potassium carbonate (K_2_CO_3_). The electrical conductivity (EC) of the water was kept at 1,000–2,000 µS cm**^−1^** by a water exchange rate of 900–1,200 L/week. All effluent water from the RAS was used to produce a RO concentrate intended as fertilizer, hereby called RAS nutrient concentrate (RAS NC). The water treatment system consisted of ultra filtration (UF) with flocculation as a pretreatment step, followed by RO.

### 2.2. Pasteurization, storage and sampling

Two 10-liter polyethylene containers were washed, disinfected with 96% ethanol, and labelled “control” and “pasteurization”. Approximately 8 liters of RO concentrate were transferred to each container via a faucet installed on the RO concentrate storage tank. Following sampling, the pasteurization sample was pasteurized by heating to 80 °C for 10 minutes in a heating cabinet, to eliminate vegetative cells [13]. Both samples were covered in opaque wrapping to eliminate light exposure and stored at room temperature (20 ± 3 °C). To determine storage stability, samples of 200 mL were taken from both treatments after 1, 6, 15, 61, 123, and 183 days of storage, by mixing the containers thoroughly before aseptically pouring RAS NC into sampling containers for water composition and microbiological analysis. Samples for visual characterization were collected using the same procedure after 1 and 183 days of storage.

### 2.3. Water composition and microbiological analysis

Water composition analysis (n = 3) of the RAS NC was performed by Eurofins (Nutrient solution analysis, test code 510, Eurofins Agro Testing Norway AS, Moss, Norway) and included the following parameters: Electrical conductivity (EC), pH, bicarbonate (HCO3), chlorine (Cl), nitrate-nitrogen (NO_3_-N), ammonium-nitrogen (NH_4_-N), phosphate (P), potassium (K), sulfur (S), calcium (Ca), magnesium (Mg), sodium (Na), iron (Fe), copper (Cu), manganese (Mn), zinc (Zn), boron (B), molybdenum (Mo), and silicon (Si).

Microbiological analysis (n = 1) was performed by Kytos (Kytos, Zwijnaarde, Belgium) to determine viable cell load, the percentage of intact bacterial cells, and bacterial diversity by flow cytometry. Samples of 1 mL were transferred into a non-transparent 2 mL vial (Kytovial, Kytos) containing glutaraldehyde fixative solution and stored at 4 °C prior to shipment under cooled (4–8 °C) conditions to Kytos Belgium. Upon arrival, the samples were diluted in 0.2 µm filtered phosphate buffered saline 1x (Merck) and stained with 1% (v/v) nucleic acid SYBR® Green I (final concentration 1:10,000) for total cell counts. For intact cell counts, a combination of Propidium Iodide and SYBR® Green I was used (final concentration 1:10,000 SYBR® Green I and 6 μM Propidium Iodide). Samples were incubated at 37 °C for 20 minutes to allow the stains to permeabilize the cells [14]. After the incubation step, measurements were performed with an Attune NxT flow cytometer (Thermo Fisher Scientific, Belgium). Stained cells were excited at 488 nm using an acquisition volume of 100 µL. To separate bacterial signals from background noise and to distinguish intact versus dead cells, fixed gates were applied as defined in [14] Van Nevel et al. (2013). The processed data were used to calculate total cell concentration, percentage of intact cells, bacterial alpha diversity, following the data processing method described in [15] Props et al. (2016).

### 2.4. Statistics

For the microbiological data analysis, raw experimental data were collected using the Attune™ Cytometric Software v. 6.2.1. Data processing was performed using Kytos’ proprietary data analysis software KytoFlow v. 1.9.8 and statistical analyses were conducted using the R language for statistical computing, v. 4.4.0. All other data and figures were processed in Microsoft Excel (version 2502). A 2-tailed *t-*test assuming equal variance with a significance level of α = 0.05 was used to test significant differences between means. All results were presented as mean values ± standard error. Experimental data have been made available in the OSF depository for the study [16].

## 3. Results and discussion

### 3.1. Physiochemical stability of pasteurized and unpasteurized RAS nutrient concentrate during 6 months of storage

**Fig 1:**
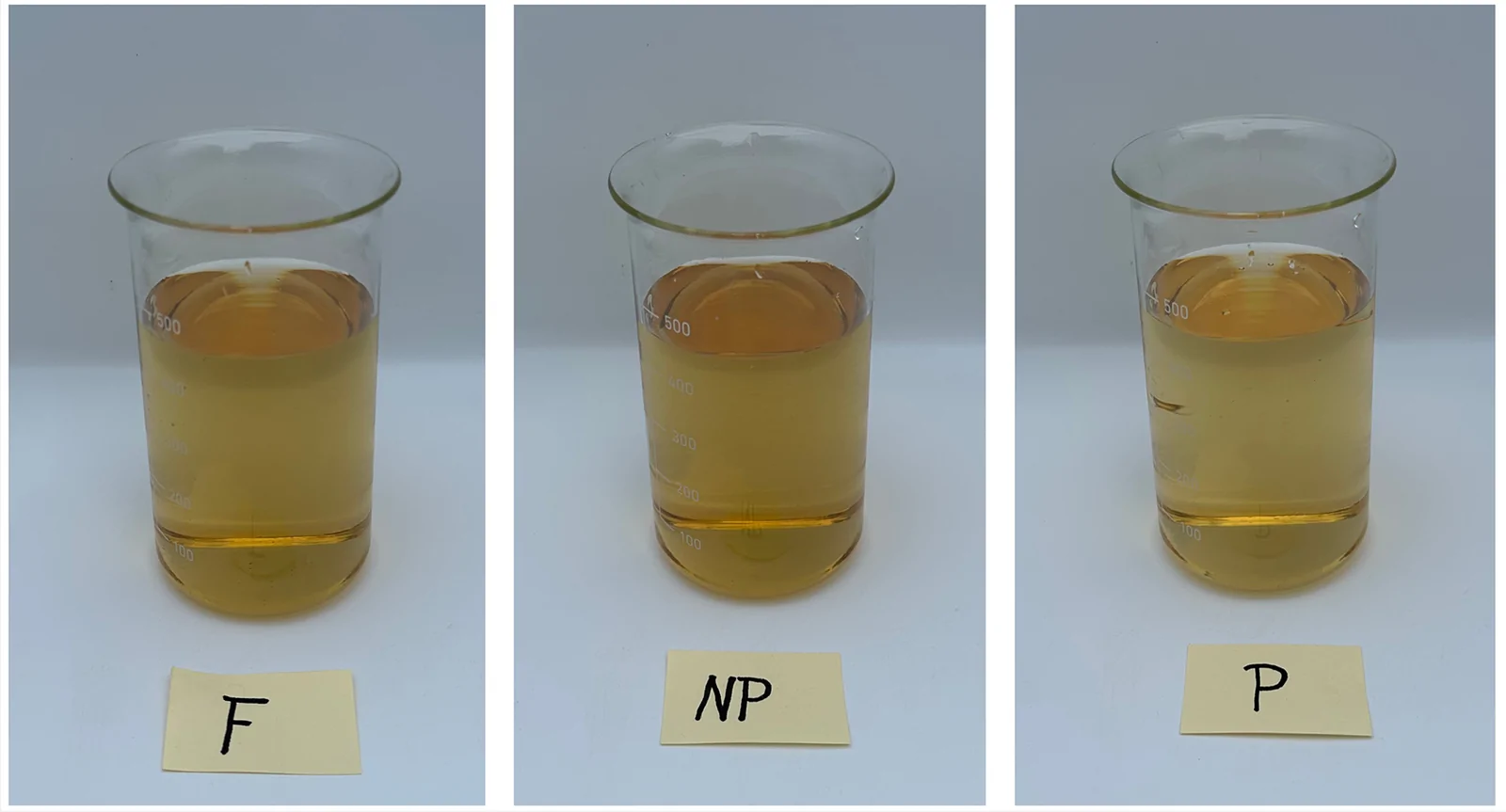
Visual characteristic of RAS NC. Left – newly produced RAS NC (F); middle – unpasteurized RAS NC after 6 months storage (NP); right – pasteurized RAS NC after 6 months storage (P). RAS NC = Recirculating aquaculture system nutrient concentrate.

Visual assessment revealed minimal changes in the RAS NC during the storage period, regardless of pasteurization (Fig 1). Both newly produced RAS NC (F) and the unpasteurized RAS NC (NP) stored for 6 months displayed the same clear, yellow color with no detectable change in odor, precipitation, or visible microbiological growth. Meanwhile, the pasteurized treatment had a slightly lighter color than both the freshly produced RAS NC and stored, unpasteurized RAS NC.

Pasteurization significantly increased the pH, decreased the EC, and decreased the concentration of plant macronutrients such as nitrate and potassium in the RAS NC (Table 1). The largest reduction was observed in iron, which decreased by 28.7%, from 112.67 ± 1.15 to 80.33 ± 0.58 µM, possibly explaining the lighter yellow colour observed in the pasteurized treatment. The effect persisted throughout the storage period of 183 days.

**Table 1:** Initial concentration of pH, EC and dissolved elements in the pasteurized RAS NC and unpasteurized control before the storage period (n = 3).

	Pasteurized RAS NC	Control
**pH**	7.37 ± 0.06	6.97 ± 0.06
**EC (mS/ cm)**	6.70 ± 0.00	7.20 ± 0.00
**NH**_**4**_ **(mM)**	<0.1	<0.1
**K (mM)**	47.27 ± 1.63	51.07 ± 1.16
**Na (mM)**	5.27 ± 0.06	5.77 ± 0.12
**Ca (mM)**	1.80 ± 0.00	1.83 ± 0.06
**Mg (mM)**	2.87 ± 0.06	3.10 ± 0.10
**NO**_**3**_ **(mM)**	40.63 ± 0.06	44.13 ± 0.31
**Cl (mM)**	7.47 ± 0.06	8.13 ± 0.06
**S (mM)**	4.87 ± 0.06	5.83 ± 0.06
**P (mM)**	<0.04	<0.04
**Fe (µM)**	82.33 ± 0.58	112.67 ± 1.15
**Mn (µM)**	1.00 ± 0.00	1.20 ± 0.00
**Zn (µM)**	14.00 ± 0.00	16.00 ± 0.00
**B (µM)**	12.00 ± 0.00	12.67 ± 0.58
**Cu (µM)**	3.53 ± 0.06	4.17 ± 0.06
**Mo (µM)**	0.30 ± 0.00	0.33 ± 0.06
**Si (mM)**	0.03 ± 0.00	0.03 ± 0.00

RAS NC = Recirculating aquaculture system nutrient concentrate; EC = Electrical conductivity; K = Potassium; Na = Sodium; Ca = Calcium; Mg = Magnesium; NO_3_ = Nitrate; Cl = Chloride; S = Sulfur; P = Phosphate; Fe = Iron, Mn = Manganese; Zn = Zinc; B = Boron; Cu = Copper; Mo = Molybdenum; Si = Silicon.

Pasteurization can alter the balance of ions in a solution by inducing physical and chemical changes that affect solubility and speciation. While most highly soluble salts such as sodium- and potassium chloride remain unaffected, sparingly soluble compounds containing calcium, magnesium, and iron may precipitate due to changes in temperature, pH, and reduction-oxidation conditions [17,18]. We hypothesize that the observed increase in the solution’s pH has resulted in precipitation of iron and other dissolved ions, explaining the observed reduced EC following pasteurization. This hypothesis is supported by studies on groundwater treatment using calcium hydroxide, where pH elevation promotes the precipitation of iron and other dissolved ions, resulting in lower concentrations of these species in the dissolved phase [19].

Precipitation was not observed during the visual assessment (Fig 1), which weakens the hypothesis. This may, however, be explained by the sampling methodology, as the storage containers were thoroughly mixed before being poured into the sample glass for analysis. The mixing could have resuspended any precipitates, preventing their visual detection. The hypothesis is, however, conjectured and requires further study to validate. Nevertheless, the observed differences in pH, EC and chemical composition in the pasteurized and unpasteurized samples suggest that pasteurization might influence both the concentrations of specific ions and the overall chemical composition of the solution.

**Fig 2:**
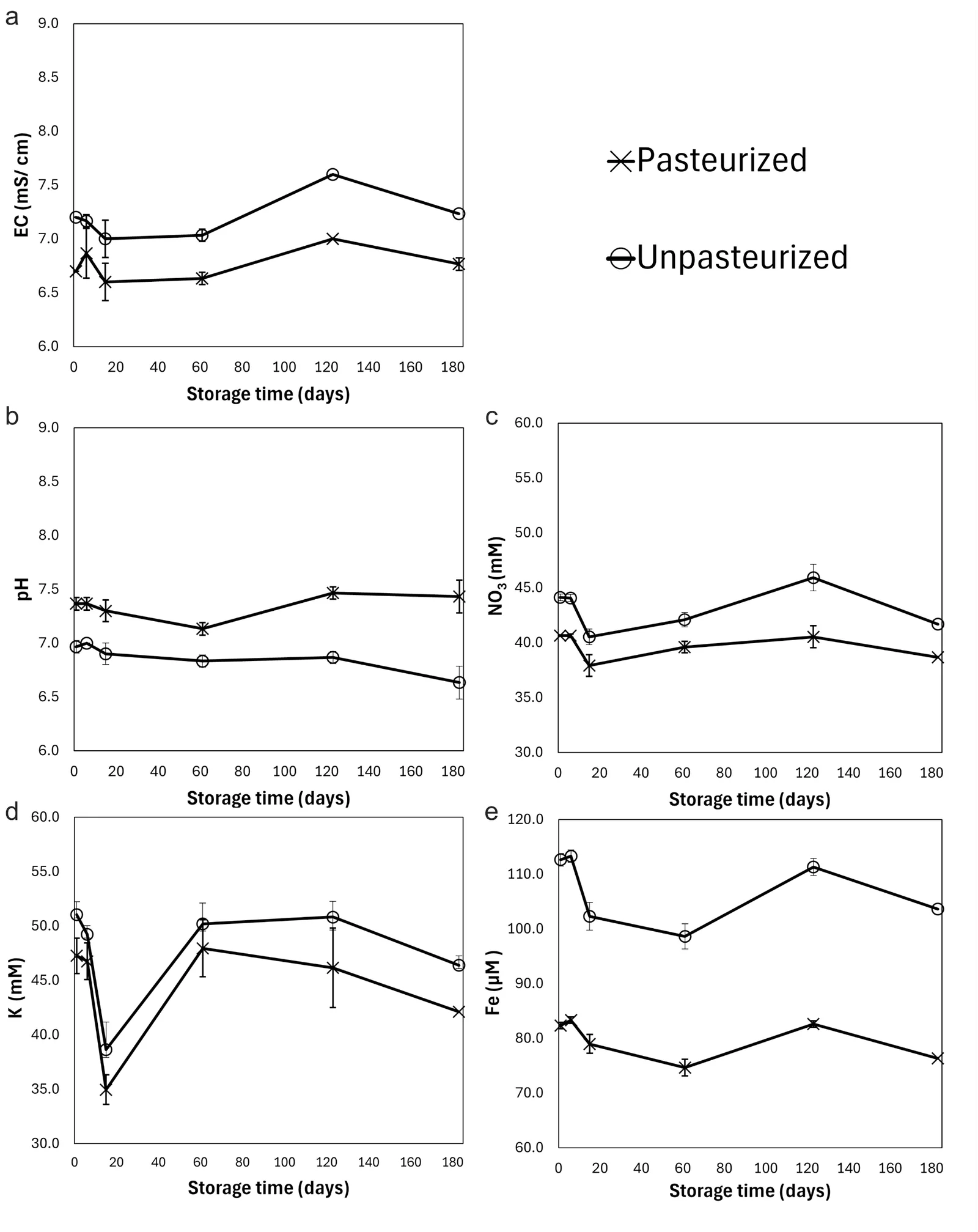
Change in chemical parameters during storage. Change in EC (a), pH (b), NO_3_ (c), K (d) and Fe (e) in pasteurized and unpasteurized RAS NC during 183 days of storage. EC = Electrical conductivity; NO_3_ = Nitrate; K = Potassium; Fe = Iron; RAS NC = Recirculating aquaculture system nutrient concentrate.

**Fig 3:**
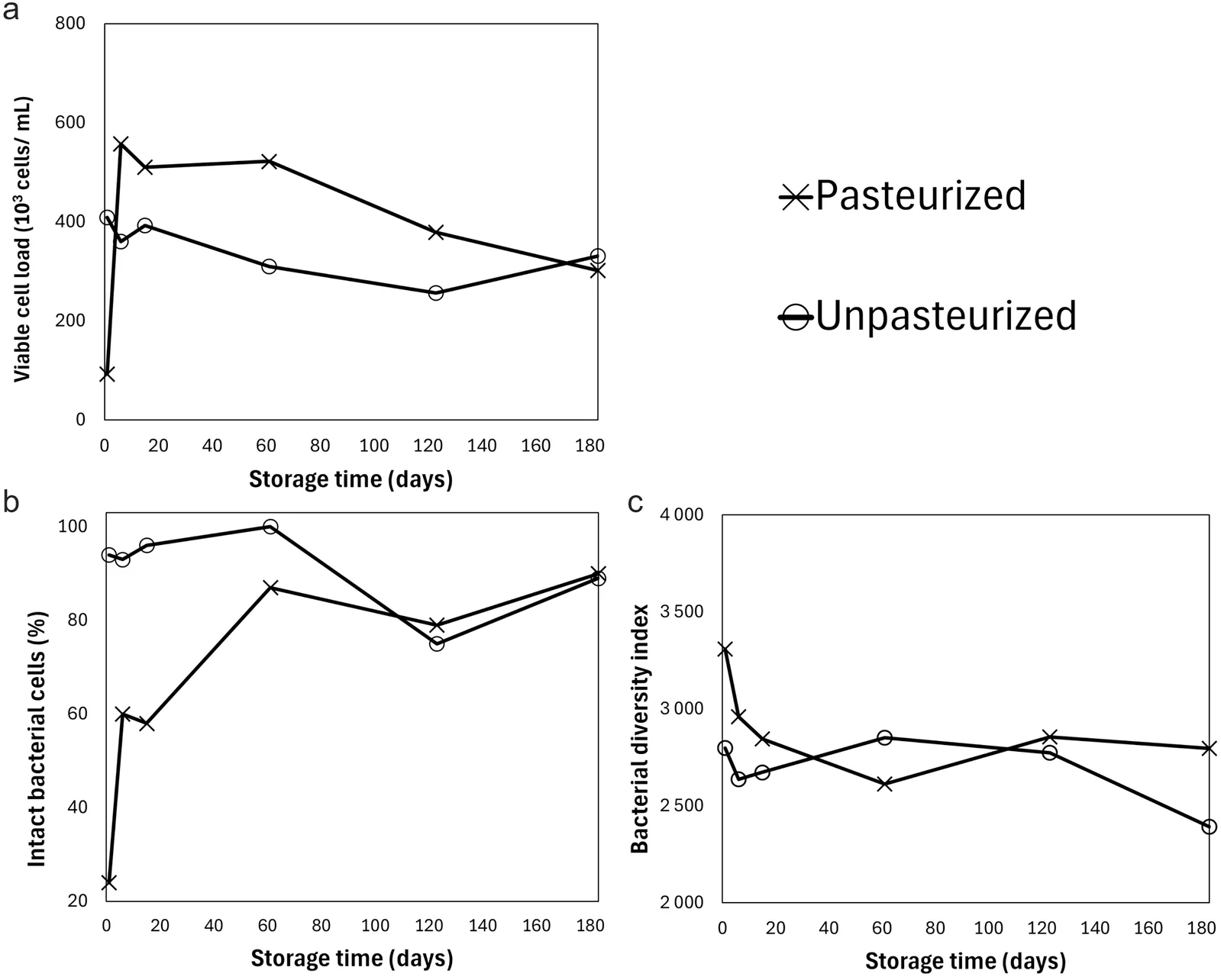
Change in microbiological parameters during storage. Viable cell load (a), intact bacterial cells (b) and bacterial diversity index (c) in pasteurized and unpasteurized RAS NC during 183 days of storage. RAS NC = Recirculating aquaculture system nutrient concentrate.

Although the EC did not change significantly from day 1 to day 183 for either treatment, it did fluctuate between the different sampling points within the storage period (Fig 2a). The observed fluctuations could be due to mineral precipitation during storage as earlier hypothesized, or due to microbiological activity absorbing nutrients. The fluctuation is especially evident in the concentrations of dissolved potassium and iron that decreased significantly from day 1 to day 15 in both treatments, before increasing again towards day 60 (Fig 2d-e). Meanwhile, the nitrate concentration displayed a slight but statistically significant decrease from day 1 to day 183 for both the pasteurized (−1.97 mM NO_3_) and unpasteurized (−2.43 mM NO_3_) treatment (Fig 2c). Finally, the pH was stable during the storage period with the exception of the unpasteurized treatment that decreased significantly (−0.23 pH) from day 123 to day 183 (Fig 2b). At the same time, the viable cell load and ratio of intact bacterial cells increased in the unpasteurized treatment towards the end of the storage period (Fig 3a and 3b, respectively). The observed reduction in pH therefore indicates increased bacterial activity linked to acid-producing metabolic processes, as described in [20].

Despite statistically significant fluctuations during storage, the EC, pH, and concentration of dissolved nutrient salts changed minimally from day 1 to day 183 of the storage period, indicating that the specific batch of RAS NC assessed in this study is functionally stable within the storage period of six months, regardless of pasteurization (Fig 2). The fluctuations of dissolved nutrient salts within the storage period, most prominent in potassium from day 1 to day 63 (Fig 2d), occurred in both treatments and were therefore not affected by pasteurization. Moreover, the heat treatment during pasteurization significantly decreased the concentration of dissolved nutrient salts, especially iron, which negatively impacts the quality of the RAS NC as a fertilizer. Combined with the increased production costs and energy requirements associated with pasteurization, it does not seem beneficial based on the physicochemical parameters.

### 3.2. Microbiological stability of pasteurized and unpasteurized RAS nutrient concentrate during 6 months of storage

Neither the pasteurized RAS NC nor the unpasteurized control showed signs of microbiological spoilage during the storage period, indicating that untreated RAS NC has a shelf life longer than six months. As expected, we observed a clear initial effect of pasteurization at day 1, where the viable cell load decreased by a factor of 4.4, from 408,900 cells/ mL in the unpasteurized treatment to 92,400 cells/ mL in the pasteurized treatment (Fig 3a). Interestingly, the viable cell load then increased again and surpassed the initial concentration in the unpasteurized treatment at day 6, before it steadily decreased towards the concentration of the unpasteurized treatment at the end of the storage period. In an enclosed system, the viable cell load is limited by a combination of resource availability, environmental conditions, and biological interactions [21]. As our containers were placed in a stable environment and shielded from light, the increasing viable cell load following pasteurization was likely driven by heterotrophic growth. Thus, the rapid recovery of viable cell load following pasteurization may have been enabled by the newly available resources released by dead cells that are then exhausted to create a stable cell population at 400,000–600,000 cells/ mL in the concentrate. Stable viable cell load over time indicates the establishment of dynamic equilibriums in the sample’s microbiological population, which in our case was briefly disrupted by the pasteurization process. While this represents an interesting observation, the establishment of stable viable cell loads independent of treatment is, in the context of shelf life, an important indicator of chemical and biological stability. The effect of pasteurization was also apparent in the percentage of intact bacterial cells (considered as live cells) where we observed a reduction from 94% to 25% after pasteurization before steadily increasing again towards the unpasteurized treatment with an interpolated intersection point after 108 days of storage (Fig 3b).

Throughout the storage period, both the viable cell load ranging from 256,500 to 408,900 cells/ mL and the bacterial diversity index ranging from 2,392 to 2,851 in the unpasteurized RAS NC remained stable (Fig 3c). In microbiological ecosystems exposed to favourable growth conditions, certain bacterial populations can rapidly dominate due to competitive advantages, resulting in an exponential growth of viable cell load [22] and a reduction in community diversity [23]. Similarly, in a spoilage scenario, we would expect dominant spoilage organisms to outcompete others, reducing overall diversity. Therefore, the lack of exponential growth in the viable cell load or a reduction in the bacterial diversity index indicates that the unpasteurized RAS NC remained microbiologically stable throughout the storage period. However, the viable cell load increased slightly in the unpasteurized control towards the end of the storage period (Fig 3a), accompanied by a decreasing bacterial diversity index (Fig 3c), which may indicate the onset of spoilage. Future studies should therefore investigate the effect of longer storage durations to observe the microbiological stability over time in concentrated organic fertilizers.

The apparent stability of the RAS NC independent of pasteurization may have been promoted by the UF pretreatment before RO. Ultrafiltration is a well-established technology used to reduce the load of microorganisms by providing a physical barrier that retains cells larger than the filter pore size of 0.01 µm. When combined with flocculation, UF also removes organic compounds that supports microbiological growth, enhancing the stability of the UF permeate. For instance, Wolf et al. (2005) [24] reported that UF was able to reduce the bacterial load by more than a 5-log reduction and the virus load by more than a 4-log reduction. Similarly, [25] Cordier et al. (2020) concluded through industrial scale experiments that UF is efficient in removing bacteria, viruses and microalgae from seawater intended for shellfish production. Pre-treating the RAS water with UF before RO, in combination with storing the RAS NC in darkness to prevent phototrophic microalgae growth, is therefore sufficient to produce a solution which is storage stable for six months independent of pasteurization.

## 4. Conclusion

The current study examined the storage stability of RO concentrate produced from RAS effluent water over a six-month storage period, with and without pasteurization. The RAS NC was stable when stored at room temperature without light for six months, regardless of pasteurization. Pasteurization did not substantially influence physical characteristics such as odor or color, and decreased the concentration of dissolved nutrient salts. Although pasteurization reduced the initial viable cell load, the microbiological population rapidly recovered to the levels of the unpasteurized treatment. Finally, an increase in the viable cell load and a decrease in the bacterial diversity index towards the end of the storage period might indicate the onset of spoilage in the unpasteurized treatment. These results indicate that untreated RAS NC is storage stable under the tested conditions, making pasteurization obsolete for storage periods less than six months. The observed stability of unpasteurized RAS NC is likely due to the pre-treatment with ultrafiltration before RO, which effectively removes microorganisms from the RAS effluent water.

This study supports nutrient recovery from RAS effluent using RO, by demonstrating that the concentrate is storage stable for up to six months independent of the energy-intensive pasteurization heat treatment. As this study relied on a single source of RAS wastewater, future studies should include multiple sources of RAS wastewater and different batches of RO concentrate to further validate the findings. Moreover, future studies should extend the storage period to evaluate the final shelf life without pasteurization, especially since the decrease in pH and increase in viable cell load towards the end of storage in the unpasteurized control may indicate the onset of spoilage. The content of nitrate in the concentrate remained stable during the storage period, indicating that RO concentrate derived from salmon RAS can provide a storage-stable, nitrogen-rich fertilizer for plant cultivation. By balancing or enriching the concentrate with organic or mineral fertilizers, the RO concentrate produced in this study could be tailored to meet the nutrient requirements of different crops, for instance, phosphorus must be added to produce a conventional NPK fertilizer. Alternatively, the concentrate may be used as a nitrogen-rich fertilizer in regions where phosphorus application is limited or restricted due to high soil phosphorus levels. Future studies should therefore conduct plant cultivation trials in which nutrient concentrate from RAS effluent replaces or supplements mineral fertilizers. To highlight the potential functional benefits of organic fertilizers in crop production, future investigations should also characterize and quantify the effects of RAS NC on soil organic carbon, microbiological diversity, and overall soil health.

## Supporting information

S1 File Graphical abstract. (TIF)
