# Supplementary material for: Liquid fertilizer recovered from the effluent water of recirculating aquaculture system by reverse osmosis exhibits storage stability independent of pasteurization
Source: PLoS One. 2026 Aug 21;21(8):e0355602. doi: 10.1371/journal.pone.0355602 (PMC13496420; doi:10.1371/journal.pone.0355602)

PLOS ONE

Liquid fertilizer recovered from the effluent water of recirculating aquaculture system by reverse osmosis exhibits storage stability independent of pasteurization

--Manuscript Draft--

**Manuscript Number:**	PONE-D-25-37522
**Article Type:**	Research Article
**Full Title:**	Liquid fertilizer recovered from the effluent water of recirculating aquaculture system by reverse osmosis exhibits storage stability independent of pasteurization
**Short Title:**	Inorganic nutrient concentrate from effluent is stable during storage independent of pasteurization
**Corresponding Author:**	Didrik Ulleberg<br>SINTEF<br>Tromsø, NORWAY
**Keywords:**	Nutrient recovery, organic fertilizer, RAS, shelf life, circularity, climate resilience, RO concentrate
**Abstract:**	The shift to a circular bioeconomy is dependent on green-tech innovations that upcycle waste streams into reusable components. The current study explores the chemical and microbiological stability of a fertilizer concentrate produced from effluent water of recirculating aquaculture systems (RAS) using reverse osmosis. The objective was to assess the shelf life of the concentrate and determine whether pasteurization is required to prevent spoilage during storage in light-proof containers at room temperature for six months. The chemical and microbial storage stability of the pasteurized concentrate was monitored by periodic sampling and compared to an untreated control. Pasteurization decreased the initial concentration of elements dissolved in the concentrate, the viable cell load and the percentage of intact bacterial cells. However, both the pasteurized and the untreated control maintained chemical and microbiological stability during storage, indicating that the concentrate has a shelf life of at least 6 months independent of pasteurization. The concentrate is therefore suitable for long-term storage and use as an organic fertilizer, without the increased production costs and energy use required for pasteurization, thereby providing a reliable and circular source of plant nutrients that can support sustainable farming practices. This study will contribute to the continued transition towards sustainable aquaculture by repurposing RAS effluent as a valuable by-product for crop cultivation.
**Order of Authors:**	Didrik Ulleberg
	Gema Raspati
	Tara Baele
	Mari Birkeland
	Jon Brage Svenning
**Additional Information:**	
**Question**	**Response**
**Financial Disclosure**<br><br>Enter a financial disclosure statement that describes the sources of funding for the work included in this submission. Review the submission guidelines for detailed requirements. View published research articles from *PLOS ONE* for specific examples.	Yes

This statement is required for submission and **will appear in the published article** if the submission is accepted. Please make sure it is accurate.<br><br>**Funded studies**<br>Enter a statement with the following details:<br>• Initials of the authors who received each award<br>• Grant numbers awarded to each author<br>• The full name of each funder<br>• URL of each funder website<br>• Did the sponsors or funders play any role in the study design, data collection and analysis, decision to publish, or preparation of the manuscript?<br><br>Did you receive funding for this work?	
Please add funding details.<br>as follow-up to "**Financial Disclosure**<br><br>Enter a financial disclosure statement that describes the sources of funding for the work included in this submission. Review the submission guidelines for detailed requirements. View published research articles from *PLOS ONE* for specific examples.<br><br>This statement is required for submission and **will appear in the published article** if the submission is accepted. Please make sure it is accurate.<br><br>**Funded studies**<br>Enter a statement with the following details:<br>• Initials of the authors who received each award<br>• Grant numbers awarded to each author<br>• The full name of each funder<br>• URL of each funder website<br>• Did the sponsors or funders play any role in the study design, data collection and analysis, decision to publish, or preparation of the manuscript?	This research is part of the IPN Nutricycle project and is funded by the Research Council of Norway (grant number # 336971). MB received salary from Columbi Farms AS and TB received salary from Kytos BV.<br>The funder had no role in study design, data collection and analysis, decision to publish, or preparation of the manuscript.

Did you receive funding for this work?"	
Please select the country of your main research funder (please select carefully as in some cases this is used in fee calculation).<br>as follow-up to "**Financial Disclosure**<br><br>Enter a financial disclosure statement that describes the sources of funding for the work included in this submission. Review the submission guidelines for detailed requirements. View published research articles from *PLOS ONE* for specific examples.<br><br>This statement is required for submission and **will appear in the published article** if the submission is accepted. Please make sure it is accurate.<br><br>**Funded studies**<br>Enter a statement with the following details:<br>• Initials of the authors who received each award<br>• Grant numbers awarded to each author<br>• The full name of each funder<br>• URL of each funder website<br>• Did the sponsors or funders play any role in the study design, data collection and analysis, decision to publish, or preparation of the manuscript?<br><br>Did you receive funding for this work?"	NORWAY - NO
**Competing Interests**<br><br>Use the instructions below to enter a competing interest statement for this submission. On behalf of all authors, disclose any competing interests that could be perceived to bias this work—acknowledging all financial support and any other relevant financial or non-financial competing interests.<br><br>This statement is required for submission and **will appear in the published article** if	I have read the journal's policy and the authors of this manuscript have the following competing interests: Coauthor MB are employed by Columbi Farms AS who has financial interests in the technology developed through the IPN Nutricycle project. All data interpretation and research findings were conducted independently and quality assured by SINTEF to ensure compliance with PLOS one guidelines. The other authors declare no competing interests.

the submission is accepted. Please make sure it is accurate and that any funding sources listed in your Funding Information later in the submission form are also declared in your Financial Disclosure statement.<br><br>View published research articles from *PLOS ONE* for specific examples.<br><br>**NO authors have competing interests**<br><br>Enter: *The authors have declared that no competing interests exist.*<br><br>**Authors with competing interests**<br><br>Enter competing interest details beginning with this statement:<br><br>*I have read the journal's policy and the authors of this manuscript have the following competing interests: [insert competing interests here]*<br><br>* typeset	
**Ethics Statement**<br><br>Enter an ethics statement for this submission. This statement is required if the study involved:<br><br>• Human participants<br>• Human specimens or tissue<br>• Vertebrate animals or cephalopods<br>• Vertebrate embryos or tissues<br>• Field research<br><br>Write "N/A" if the submission does not require an ethics statement.<br><br>General guidance is provided below. Consult the submission guidelines for detailed instructions. **Make sure that all information entered here is included in the Methods section of the manuscript.**	N/A

**Format for specific study types**<br><br>**Human Subject Research (involving human participants and/or tissue)**<br>• Give the name of the institutional review board or ethics committee that approved the study<br>• Include the approval number and/or a statement indicating approval of this research<br>• Indicate the form of consent obtained (written/oral) or the reason that consent was not obtained (e.g. the data were analyzed anonymously)<br><br>**Animal Research (involving vertebrate animals, embryos or tissues)**<br>• Provide the name of the Institutional Animal Care and Use Committee (IACUC) or other relevant ethics board that reviewed the study protocol, and indicate whether they approved this research or granted a formal waiver of ethical approval<br>• Include an approval number if one was obtained<br>• If the study involved *non-human primates*, add *additional details* about animal welfare and steps taken to ameliorate suffering<br>• If anesthesia, euthanasia, or any kind of animal sacrifice is part of the study, include briefly which substances and/or methods were applied<br><br>**Field Research**<br><br>Include the following details if this study involves the collection of plant, animal, or other materials from a natural setting:<br>• Field permit number<br>• Name of the institution or relevant body that granted permission	
**Data Availability**<br><br>Authors are required to make all data underlying the findings described fully available, without restriction, and from the time of publication. PLOS allows rare exceptions to address legal and ethical concerns. See the PLOS Data Policy and FAQ for detailed information.	Yes - all data are fully available without restriction

A Data Availability Statement describing where the data can be found is required at submission. Your answers to this question constitute the Data Availability Statement and **will be published in the article**, if accepted.<br><br>**Important:** Stating 'data available on request from the author' is not sufficient. If your data are only available upon request, select 'No' for the first question and explain your exceptional situation in the text box.<br><br>Do the authors confirm that all data underlying the findings described in their manuscript are fully available without restriction?	
**Describe where the data may be found in full sentences. If you are copying our sample text, replace any instances of XXX with the appropriate details.**<br><br>• If the data are **held or will be held in a public repository**, include URLs, accession numbers or DOIs. If this information will only be available after acceptance, indicate this by ticking the box below. For example: *All XXX files are available from the XXX database (accession number(s) XXX, XXX.*).<br>• If the data are all contained **within the manuscript and/or Supporting Information files**, enter the following: *All relevant data are within the manuscript and its Supporting Information files.*<br>• If neither of these applies but you are able to provide **details of access elsewhere**, with or without limitations, please do so. For example:<br><br>*Data cannot be shared publicly because of [XXX]. Data are available from the XXX Institutional Data Access / Ethics Committee (contact via XXX) for researchers who meet the criteria for access to confidential data.*<br><br>*The data underlying the results presented in the study are available from (include the name of the third party*	All underlying data are available from the OSF data repository through the following URL:<br>https://osf.io/9bqpc/?view_only=b61606c3dd524ce3a9a3692b7dd7c81e

and contact information or URL).<br>• This text is appropriate if the data are owned by a third party and authors do not have permission to share the data.<br><br>* typeset	
Additional data availability information:	Tick here if the URLs/accession numbers/DOIs will be available only after acceptance of the manuscript for publication so that we can ensure their inclusion before publication.

Powered by Editorial Manager® and ProduXion Manager® from Aries Systems Corporation

Cover Letter Click here to access/download;Manuscript;Manuscript.docx

Liquid fertilizer recovered from the effluent water of recirculating aquaculture system by reverse osmosis exhibits storage stability independent of pasteurization

Didrik Ulleberg*[1], Gema Raspati[2], Tara Baele[3], Mari Birkeland[4], Jon Brage Svenning[1]

[1]SINTEF Nord, Storgata 118, 9008 Tromsø, Norway
[2]Group of Water and Environment, SINTEF Community, S.P. Andersens vei 3, 7034 Trondheim, Norway
[3]Kytos, Zwijnaarde, 9052, Belgium
[4]Columbi Farms, Stokksundveien 1376, 7177 Refsnes, Norway

*Corresponding author
E-mail: didrik.ulleberg@sintef.no (DU)

Abstract

The shift to a circular bioeconomy is dependent on green-tech innovations that upcycle waste streams into reusable components. The current study explores the chemical and microbiological stability of a fertilizer concentrate produced from effluent water of recirculating aquaculture systems (RAS) using reverse osmosis. The objective was to assess the shelf life of the concentrate and determine whether pasteurization is required to prevent spoilage during storage in light-proof containers at room temperature for six months. The chemical and microbial storage stability of the pasteurized concentrate was monitored by periodic sampling and compared to an untreated control. Pasteurization decreased the initial concentration of elements dissolved in the concentrate, the viable cell load and the percentage of intact bacterial cells. However, both the pasteurized and the untreated control maintained chemical and microbiological stability during storage, indicating that the concentrate has a shelf life of at least 6 months independent of pasteurization. The concentrate is therefore suitable for long-term storage and use as an organic fertilizer, without the increased production costs and energy use required for pasteurization, thereby providing a reliable and circular source of plant nutrients that can support sustainable farming practices. This study will contribute to the continued transition towards sustainable aquaculture by repurposing RAS effluent as a valuable by-product for crop cultivation.

1. Introduction

Modern agriculture is dependent on mineral fertilizers to produce sufficient food for the still-growing world population. Norway is no exception; according to Statistics Norway, a total of 87,000 tons of nitrogen and 7,000 tons of phosphorus were utilized from mineral fertilizers in 2023 [1]. The reliance on mineral fertilizers for agriculture represents a major concern in the global food production system. The production of nitrogen mineral fertilizer requires hydrogen produced from natural gas, in addition to being an energy-intensive process that accounts for around 2% of the global energy consumption and 450 Mt CO_2 emissions annually [2]. Meanwhile, the production of phosphorus mineral fertilizer is dependent on gradually declining reserves of phosphate rock exported from deposits concentrated in Morocco and Western Sahara [3]. Therefore, the fertilizer market is subject to unpredictable pricing and availability due to the increasingly unstable energy market and rising geopolitical tensions worldwide.

At the same time, Norwegian aquaculture releases vast quantities of organic and inorganic nutrients into the ocean as waste. A 2021 study estimated that Norwegian mariculture released 60,000 tons of nitrogen and 14,000 tons of phosphorus annually [4]. Most of these nutrient salts are released directly into the ocean through open net-pen production and are therefore unavailable for nutrient recovery, with the exception of locations with infrastructure for sludge collection [5]. However, increased production of Atlantic salmon (*Salmo salar)* and rainbow trout (*Oncorhynchus mykiss*) in recirculating aquaculture systems (RAS) could enable nutrient recovery from water and sludge waste streams [6]. In 2020, land-based

aquaculture facilities in Norway were estimated to release 925 tons of nitrogen and 149 tons of phosphate annually [4].

Recirculating aquaculture systems are land-based fish cultivation facilities characterized by a high degree of water reuse [7]. To maintain optimal water quality in the rearing tanks, water is circulated through several treatment steps such as mechanical filtration, oxygenation, CO_2 degassing, pH adjustment, sludge removal, and biofilters. Within the biofilter, ammonia excreted by the fish is converted to nitrite and then to nitrate by nitrifying bacteria, resulting in nitrate accumulation over time. Therefore, some water exchange is necessary to prevent nutrient salts from accumulating to toxic levels. The resulting effluent water is sometimes treated with biological denitrification to reduce eutrophication before it is discarded [8], and suspended solids and sludge are removed by filtration. To increase the sustainability of land-based aquaculture, nutrient salts accumulated in RAS effluent water should ideally be recovered and used as plant fertilizer. However, there are currently no technologies implemented in commercial scale facilities that enable nutrient recovery. Thus, the transition to a circular bioeconomy will require novel solutions that enable nutrient recovery from waste streams, thereby alleviating the consumption of mineral fertilizer.

Although nitrate accumulates in the water of RAS during recirculation, its final concentration remains low compared to that of commercial liquid fertilizers [9]. The water could be used directly for fertilizer in aquaponic cultivation systems, but in most cases the nutrient concentration is too low to support optimal plant growth without supplementation with mineral fertilizer [10]. Furthermore, the amount of mineral salts generated in a full-scale RAS in Norway exceeds the fertilizer requirements of local farms, necessitating transport to other locations. Therefore,

widespread utilization of RAS effluent water as a plant fertilizer is dependent on water-treatment technologies, such as reverse osmosis (RO), that concentrate nutrient salts for efficient transportation, storage, and distribution, while simultaneously producing clean water that can increase the degree of water recirculation in RAS.

Finally, the commercial viability of any organic fertilizer depends on its storage stability, as these products are typically acquired in bulk and utilized gradually over the duration of a growth season. Untreated organic fertilizers may be susceptible to spoilage during storage due to their high water activity and organic load, either by microbial growth of algae, fungi, and bacteria, or by chemical changes including precipitation, oxidation, and enzymatic reactions [11]. Controlling storage conditions such as ambient temperature, pH, oxygen availability, and light exposure is therefore essential to preserve nutrient availability, and to stabilize the microbial activity in the product. Conventional preservative methods such as pasteurization may also be necessary to improve the storage stability by inhibiting the growth of microorganisms [12].

In the current study, a RO system was used to recover plant nutrients from salmon RAS effluent water. The aim of the study was to monitor the storage stability of the resulting RO concentrate over six months, with and without pasteurization. By demonstrating how underutilized organic waste streams can be converted into storage-stable nutrient concentrates, the study promotes holistic approaches to food production and can contribute to reducing the use of mineral fertilizers in crop production.

2. Materials and methods

2.1. Production of Nutrient Concentrate from RAS effluent water

RAS effluent water was sourced from a research-scale RAS facility at NIBIO Landvik, Norway. Salmon (*Salmo salar)* were reared in 1,000 L tanks at a density between 70–100 kg/m³ and fed with Nutra RC® feed (Skretting, Stavanger, Norway). The RAS system included two fish rearing tanks, a moving bed biofilter to convert ammonia to nitrate, and a hydrocyclone in combination with a drum filter for removal of faeces and particles. The oxygen level was kept at 100% saturation, with the use of pure oxygen in nanobubbles, while CO_2 was removed from the water by a CO_2 scrubber. The pH was kept at 6.85 by buffering with potassium carbonate (K_2CO_3). The electrical conductivity (EC) of the water was kept at 1,000–2,000 µS cm^{-1} by a water exchange rate of 900–1,200 L/week. All effluent water from the RAS was used to produce a RO concentrate intended as fertilizer, hereby called RAS nutrient concentrate (RAS NC). The water treatments system consisted of ultra filtration (UF) with flocculation as a pretreatment step, followed by RO.

2.2. Pasteurization, storage and sampling

Two 10-liter polyethylene containers were washed, disinfected with 96% ethanol, and labelled “control” and “heat treatment”. Approximately 8 liters of RO concentrate were transferred to each container via a faucet installed on the RO concentrate storage tank. Following sampling, the heat treatment sample was heated to 80 °C for 10 minutes in a heating cabinet. Both samples were covered in black plastic to

reduce light exposure and placed in the dark at room temperature (20 ± 3 °C) for storage immediately after sampling (control) and pasteurization (heat treatment). To determine storage stability, analytical samples of 200 mL were taken from both treatments after 1, 6, 15, 61, 123, and 183 days of storage, by mixing the containers thoroughly before aseptically pouring RAS NC into sampling containers for water composition and microbiological analysis.

2.3. Water composition and microbiological analysis

Water composition analysis (n=3) of the RAS NC was performed by Eurofins (Nutrient solution analysis, test code 510, Eurofins Agro Testing Norway AS, Moss, Norway) and included the following parameters: Electrical conductivity (EC), pH, bicarbonate (HCO3), chlorine (Cl), nitrate-nitrogen (NO_3-N), ammonium-nitrogen (NH_4-N), phosphate (P), potassium (K), sulfur (S), calcium (Ca), magnesium (Mg), sodium (Na), iron (Fe), copper (Cu), manganese (Mn), zinc (Zn), boron (B), molybdenum (Mo), and silicon (Si).

Microbiological analysis (n=1) was performed by Kytos (Kytos, Zwijnaarde, Belgium) to determine viable cell load, the percentage of intact bacterial cells, and bacterial diversity by flow cytometry. Samples of 1 mL were transferred into a non-transparent 2 mL vial (Kytovial, Kytos) containing glutaraldehyde fixative solution and stored at 4 °C prior to shipment under cooled (4–8 °C) conditions to Kytos Belgium. Upon arrival, the samples were diluted in 0.2 µm filtered phosphate buffered saline 1x (Merck) and stained with 1% (v/v) nucleic acid SYBR® Green I (final concentration 1:10,000) for total cell counts. For intact cell counts, a combination of Propidium

Iodide and SYBR® Green I was used (final concentration 1:10,000 SYBR® Green I and 6 µM Propidium Iodide). Samples were incubated at 37 °C for 20 minutes to allow the stains to permeabilize the cells [13]. After the incubation step, measurements were performed with an Attune NxT flow cytometer (Thermo Fisher Scientific, Belgium). Stained cells were excited at 488 nm using an acquisition volume of 100 µL. To separate bacterial signals from background noise and to distinguish intact versus dead cells, fixed gates were applied as defined in [13] Van Nevel et al. (2013). The processed data were used to calculate total cell concentration, percentage of intact cells, bacterial alpha diversity, following the data processing method described in [14] Props et al. (2016).

2.4. Statistics

For the microbiological data analysis, raw experimental data were collected using the Attune™ Cytometric Software v. 6.2.1. Data processing was performed using Kytos' proprietary data analysis software KytoFlow v. 1.9.8 and statistical analyses were conducted using the R language for statistical computing, v. 4.4.0. All other data and figures were processed in Microsoft Excel (version 2502). A 2-tailed *t*-test assuming equal variance with a significance level of $\alpha = 0.05$ was used to test significant differences between means. All results were presented as mean values ± standard error. Experimental data have been made available in the OSF depository for the study [15].

3. Results and discussion

3.1. Physiochemical stability of pasteurized and unpasteurized RAS nutrient concentrate during 6 months of storage

Visual assessment of physical characteristics revealed minimal changes in the RAS NC during the storage period, regardless of pasteurization (Fig 1). Both newly produced RAS NC (F) and the unpasteurized RAS NC (NP) stored for 6 months displayed the same clear, yellow color with no detectable change in odor, precipitation, or visible microbial growth. Meanwhile, the pasteurized treatment had a slightly lighter color than both the freshly produced RAS NC and stored, unpasteurized RAS NC.

Fig 1. Physical characteristic of RAS NC. Left - newly produced RAS NC (F); middle - unpasteurized RAS NC after 6 months storage (NP); right – pasteurized RAS NC after 6 months storage (P).

Pasteurization significantly increased the pH, decreased the EC, and decreased the concentration of plant macronutrients such as nitrate and potassium in the RAS NC (table 1). The largest reduction was observed in iron, which decreased by 28.7%, from 112.67 ± 1.15 to 80.33 ± 0.58 mM, possibly explaining the lighter yellow colour observed in the pasteurized treatment. The effect persisted throughout the storage period of 183 days.

Table 1. Initial concentration of pH, EC and dissolved elements in the pasteurized RAS NC and unpasteurized control before the storage period (n=3).

	Pasteurized RAS NC	Control
**pH**	7.37 ± 0.06	6.97 ± 0.06

**EC (mS/cm)**	6.70 ± 0.00	7.20 ± 0.00
**NH_4 (mM)**	<0.1	<0.1
**K (mM)**	47.27 ± 1.63	51.07 ± 1.16
**Na (mM)**	5.27 ± 0.06	5.77 ± 0.12
**Ca (mM)**	1.80 ± 0.00	1.83 ± 0.06
**Mg (mM)**	2.87 ± 0.06	3.10 ± 0.10
**NO_3 (mM)**	40.63 ± 0.06	44.13 ± 0.31
**Cl (mM)**	7.47 ± 0.06	8.13 ± 0.06
**S (mM)**	4.87 ± 0.06	5.83 ± 0.06
**P (mM)**	<0.04	<0.04
**Fe (µM)**	82.33 ± 0.58	112.67 ± 1.15
**Mn (µM)**	1.00 ± 0.00	1.20 ± 0.00
**Zn (µM)**	14.00 ± 0.00	16.00 ± 0.00
**B (µM)**	12.00 ± 0.00	12.67 ± 0.58
**Cu (µM)**	3.53 ± 0.06	4.17 ± 0.06
**Mo (µM)**	0.30 ± 0.00	0.33 ± 0.06
**Si (mM)**	0.03 ± 0.00	0.03 ± 0.00

RAS NC = Recirculating aquaculture system nutrient concentrate; EC = Electrical conductivity; K = Potassium; Na = Sodium; Ca = Calcium; Mg = Magnesium; NO_3 = Nitrate; Cl = Chloride; S = Sulfur; P = Phosphate; Fe = Iron, Mn = Manganese; Zn = Zinc; B = Boron; Cu = Copper; Mo = Molybdenum; Si = Silicone.

Pasteurization can alter the balance of ions in a solution by inducing physical and chemical changes that affect solubility and speciation. While most highly soluble salts such as sodium- and potassium chloride remain unaffected, sparingly soluble compounds containing calcium, magnesium, and iron may precipitate due to changes in temperature, pH, and reduction-oxidation conditions [16,17]. The loss of carbon dioxide during heating can increase the solution’s pH promoting the precipitation of carbonate and hydroxide salts of the abovementioned metals. These processes reduce the concentration of certain ions in the dissolved phase and, thereby, shifting the ionic equilibrium of the solution without necessarily altering the total mass of ions present. As a result, pasteurization can influence both the measured concentrations of specific ions and the overall chemical composition of the solution.

Although the EC did not change significantly from day 1 to day 183 for either treatment, it did fluctuate between the different sampling points within the storage period (Fig 2a). As the EC is used as an indirect measure of the concentration of nutrient salts dissolved in the solution, the observed change in EC could be due to salts that continuously precipitate and dissolve during the storage period. The fluctuation is especially evident in the concentrations of dissolved potassium and iron that decreased significantly from day 1 to day 15 in both treatments, before increasing again towards day 60 (Fig 2d-e). Meanwhile, the nitrate concentration displayed a slight but statistically significant decrease from day 1 to day 183 for both the pasteurized (-1.97 mM NO_3) and unpasteurized (-2.43 mM NO_3) treatment (Fig 2c). Finally, the pH was stable during the storage period with the exception of the unpasteurized treatment that decreased significantly (-0.23 pH) from day 123 to day 183, which may indicate increased bacterial activity [18] towards the end of the storage period in the unpasteurized RAS NC (Fig 2b).

Fig 2. Change in chemical parameters during storage. Change in EC (a), pH (b), NO_3 (c), K (d) and Fe (e) in pasteurized and unpasteurized RAS nutrient concentrate during 183 days of storage. EC = Electrical conductivity; NO_3 = Nitrate; K = Potassium; Fe = Iron.

Despite statistically significant fluctuations during storage, the EC, pH, and concentration of dissolved nutrient salts changed minimally from day 1 to day 183 of the storage period, indicating that the RAS NC is sufficiently stable during six months of storage regardless of pasteurization (Fig 2). The fluctuations of dissolved nutrient salts within the storage period, most prominent in potassium from day 1 to day 63 (Fig 2d), occurred in both treatments and were therefore not affected by pasteurization. Moreover, the heat treatment during pasteurization significantly decreased the concentration of dissolved nutrient salts, especially iron, which

negatively impacts the quality of the RAS NC as a fertilizer. Combined with the increased production costs and energy requirements associated with pasteurization, it does not seem beneficial based on the physicochemical parameters.

3.2. Microbial stability of pasteurized and unpasteurized RAS nutrient concentrate during 6 months of storage

Neither the pasteurized RAS NC nor the unpasteurized control showed signs of microbiological spoilage during the storage period, indicating that untreated RAS NC has a shelf life longer than six months. We observed a clear initial effect of pasteurization at day 1, where the viable cell load decreased by a factor of 4.4, from 408,900 cells/ mL in the unpasteurized treatment to 92,400 cells/ mL in the pasteurized treatment (Fig 3a). Interestingly, the viable cell load then increased again and surpassed the initial concentration in the unpasteurized treatment at day 6, before it steadily decreased towards the concentration of the unpasteurized treatment at the end of the storage period. The rapid recovery of viable cell load after pasteurization could be due to exponential growth enabled by newly available resources released by dead cells that are then exhausted to create a stable cell population at 400,000 – 600,000 cells/ mL in the concentrate. The effect of pasteurization is also apparent in the percentage of intact bacterial cells (considered as live cells) where we observed a reduction from 94% to 25% after pasteurization

Figure 3. Change in microbiological parameters during storage. Viable cell load (a), intact bacterial cells (b) and bacterial diversity index (c) in pasteurized and unpasteurized RAS nutrient concentrate during 183 days of storage.

before it steadily increased again towards the unpasteurized treatment with an interpolated intersection point after 108 days of storage (Fig 3b).

Throughout the storage period, both the viable cell load ranging from 256,500 to 408,900 cells/ mL and the bacterial diversity index ranging from 2,392 to 2,851 in the unpasteurized RAS NC remained stable (Fig 3c). In microbial ecosystems exposed to favorable growth conditions, certain bacterial populations can rapidly dominate due to competitive advantages, resulting in an exponential growth of viable cell load [19] and a reduction in community diversity [20]. Similarly, in a spoilage scenario, we would expect dominant spoilage organisms to outcompete others, reducing overall diversity. Therefore, the lack of exponential growth in the viable cell load or a reduction in the bacterial diversity index indicates that the unpasteurized RAS NC remained microbiologically stable throughout the storage period. However, the viable cell load slightly increase in the unpasteurized control towards the end of the storage period (Fig 3a), which may indicate the onset of spoilage that could develop into exponential growth followed by spoilage beyond the storage period of six months.

The apparent stability of the RAS NC independent of pasteurization might be caused by the UF pretreatment before RO. Ultrafiltration is a well-established technology used to reduce the load of microorganisms by providing a physical barrier that retains cells larger than the filter pore size of 0.01 µm. When combined with flocculation, UF also removes organic compounds that supports microbial growth, enhancing the stability of the UF permeate. For instance [21], Wolf et al. (2005) reported that UF was able to reduce the bacterial load by more than a 5-log reduction and the virus load by more than a 4-log reduction. Similarly, [22] Cordier et

al. (2020) concluded through industrial scale experiments that UF is efficient in removing bacteria, viruses and microalgae from seawater intended for shellfish production. Pre-treatment of the RAS water by UF before RO, combined with storage of the RAS NC under dark conditions to inhibit growth of phototrophic microalgae, therefore seems to make pasteurization obsolete for six-months storage.

4. Conclusion

The current study examined the storage stability of RO concentrate produced from RAS effluent water over a six-month storage period, with and without pasteurization. The RAS NC was stable when stored at room temperature without light for six months, regardless of pasteurization. Pasteurization did not substantially influence physical characteristics such as odor and color, and decreased the concentration of dissolved nutrient salts. Although pasteurization reduced the initial viable cell load, the microbial population rapidly recovered to the levels of the unpasteurized treatment. These results indicate that untreated RAS NC has a shelf life longer than six months, making pasteurization obsolete. The observed stability of unpasteurized RAS NC is likely due to the pre-treatment with ultrafiltration before RO, which effectively removes microorganisms from the RAS effluent water.

This study supports nutrient recovery from RAS effluent using RO, by demonstrating that the shelf life of the concentrate is longer than six months independent of the energy-intensive pasteurization heat treatment. Future studies should extend the storage period to evaluate the final shelf life without pasteurization, especially since the decrease in pH and increase in viable cell load towards the end of storage in the unpasteurized control may indicate the onset of spoilage. Moreover, future studies

should conduct plant cultivation trials in which nutrient concentrate from RAS effluent replace mineral fertilizers. To highlight the functional benefits of organic fertilizers in crop production, future investigations should also characterize and quantify the effects of RAS NC on soil organic carbon, microbial diversity, and overall soil health.

Acknowledgements

We would like to thank the team at NIBIO Landvik including Iris Eik, Atle Beisland, Olav Langmyr, Pim Vingerhoets and Randi Seljåsen, for their dedicated effort in system installation and operation as well as experimental setup and sampling. We would also like to thank Gjermund Bahr and Siv-Lene Gangenes Skar for their guidance throughout the planning and execution of the project.

References

1. Bjørlo B, Steffenstorpet R. Bruk av gjødsel i jordbruket [Internet]. Database: SSB; [cited 2025 Apr 10]. Available from: https://www.ssb.no/jord-skog-jakt-og-fiskeri/jordbruk/artikler/bruk-av-gjodsel-i-jordbruket

2. Rosa L, Gabrielli P. Energy and food security implications of transitioning synthetic nitrogen fertilizers to net-zero emissions. Environ Res Lett. 2022;18:014008.

3. Daneshgar S, Callegari A, Capodaglio AG, Vaccari D. The Potential Phosphorus Crisis: Resource Conservation and Possible Escape Technologies: A Review. Resources. 2018;7:37.

4. Øyvind Hilmarsen, Ole Jacob Broch, Ingebrigt Uglem, Matilde Skogen Chauton, Johanna Järnegren, Andreas Hagemann, et al. Kunnskaps og erfaringskartlegging om effekter av og muligheter for utnyttelse av organisk materiale og næringssalter fra havbruk [Internet]. SINTEF Nord; 2021 Jan p. 198. Report No.: 2020:01383. Available from: https://www.fhf.no/prosjekter/prosjektbasen/901572/

5. PwC, Ragn-Sells Havbruk, Framo Aquaculture, AMOF Fjell Technology, NCE Seafood Innovation. Ny grønn verdikjede [Internet]. Available from: https://www.pwc.no/no/publikasjoner/ny-groenn-verdikjede-med-slam-fra-havbruk.html

6. Dalsgaard J, Lund I, Thorarinsdottir R, Drengstig A, Arvonen K, Pedersen PB. Farming different species in RAS in Nordic countries: Current status and future perspectives. Aquac Eng. 2013;53:2–13.

7. Brown AR, Wilson ,Rod W., and Tyler CR. Assessing the Benefits and Challenges of Recirculating Aquaculture Systems (RAS) for Atlantic Salmon Production. Rev Fish Sci Aquac. 2023;0:1–22.

8. Rijn J van. Waste treatment in recirculating aquaculture systems. Aquac Eng. 2013;53:49–56.

9. Möller K, and Schultheiß U. Chemical characterization of commercial organic fertilizers. Arch Agron Soil Sci. 2015;61:989–1012.

10. Jakobsen ØM, Schiefloe M, Simonsen G, Attramadal KJK, Jost AIK. Utilization of runoff nutrients from recirculating aquaculture systems for hydroponic crop cultivation. Acta Hortic. 2021;221–8.

11. Mafe AN, Edo GI, Makia RS, Joshua OA, Akpoghelie PO, Gaaz TS, et al. A review on food spoilage mechanisms, food borne diseases and commercial aspects of food preservation and processing. Food Chem Adv. 2024;5:100852.

12. Deak T. Food Safety Management. 1st ed. Motarjemi Y, Lelieveld H, editors. San Diego: Academic Press; 2014.

13. Van Nevel S, Koetzsch S, Weilenmann H-U, Boon N, Hammes F. Routine bacterial analysis with automated flow cytometry. J Microbiol Methods. 2013;94:73–6.

14. Props R, Monsieurs P, Mysara M, Clement L, Boon N. Measuring the biodiversity of microbial communities by flow cytometry. Methods Ecol Evol. 2016;7:1376–85.

15. Ulleberg D. Concentrated fish-water displays storage stability independent of pasteurization [Internet]. OSF; 2025. Available from: https://osf.io/9bqpc/?view_only=b61606c3dd524ce3a9a3692b7dd7c81e

16. Shiratori M, Kiss R. Risk Mitigation in Preventing Adventitious Agent Contamination of Mammalian Cell Cultures. In: Kiss B, Gottschalk U, Pohlscheidt M, editors. New Bioprocess Strateg Dev Manuf Recomb Antibodies Proteins [Internet]. Cham: Springer International Publishing; 2018 [cited 2025 Jul 7]. p. 75–93. Available from: https://doi.org/10.1007/10_2017_38

17. Djemal L, Fournier C, von Hagen J, Kolmar H, Deparis V. Review: High temperature short time treatment of cell culture media and feed solutions to mitigate adventitious viral contamination in the biopharmaceutical industry. Biotechnol Prog. 2021;37:e3117.

18. Fang Z, Zhao Y, Warner RD, Johnson SK. Active and intelligent packaging in meat industry. Trends Food Sci Technol. 2017;61:60–71.

19. Steele R. Understanding and Measuring the Shelf-Life of Food. Woodhead Publishing; 2004.

20. Props R, Schmidt ML, Heyse J, Vanderploeg HA, Boon N, Denef VJ. Flow cytometric monitoring of bacterioplankton phenotypic diversity predicts high

population-specific feeding rates by invasive dreissenid mussels. Environ Microbiol. 2018;20:521–34.

21. Wolf PH, Siverns S, Monti S. UF membranes for RO desalination pretreatment. Desalination. 2005;182:293–300.

22. Cordier C, Stavrakakis C, Morga B, Degrémont L, Voulgaris A, Bacchi A, et al. Removal of pathogens by ultrafiltration from sea water. Environ Int. 2020;142:105809.

Figure 2

Click here to access/download;Figure;Fig2.tif

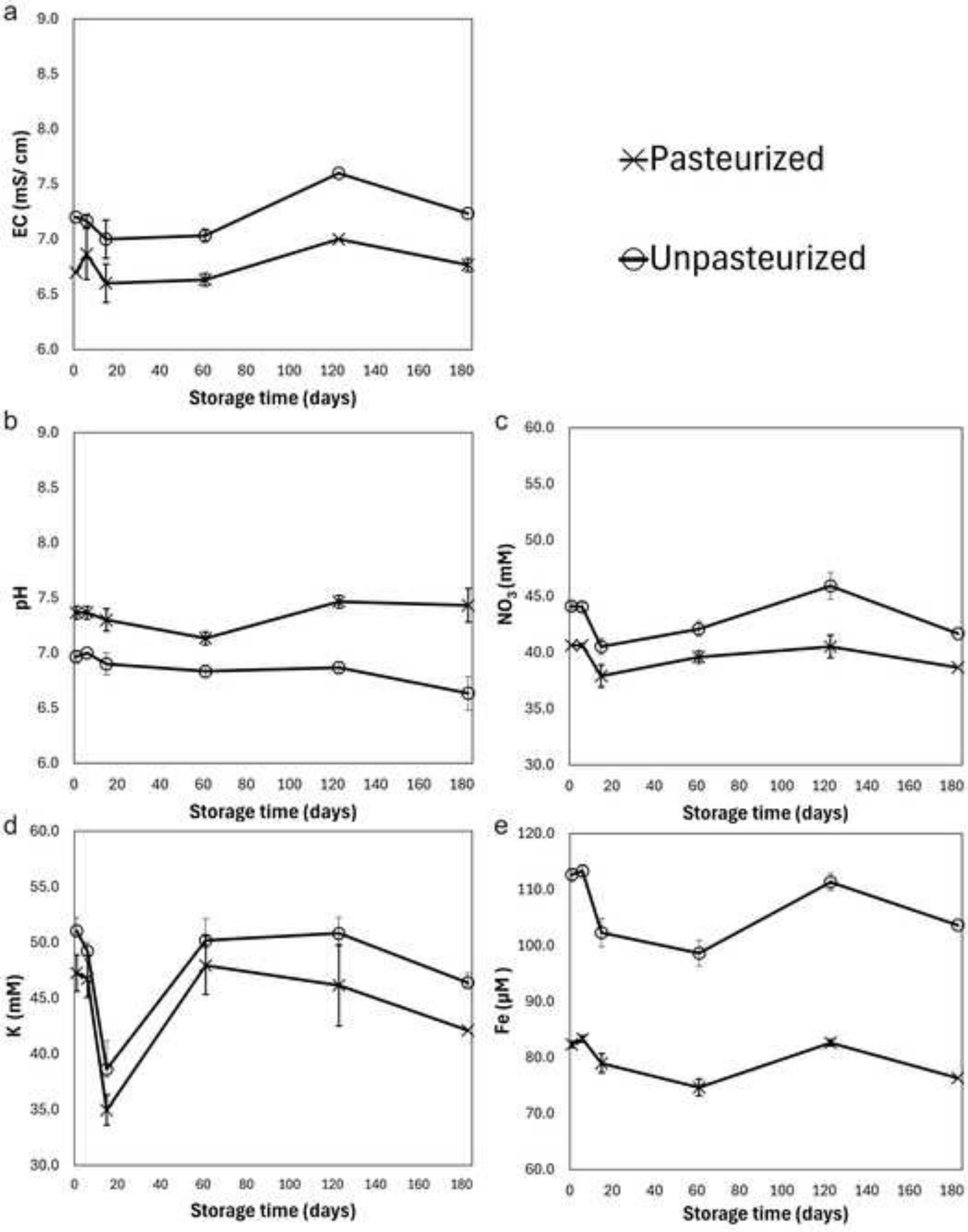

Figure 3

Click here to access/download;Figure;Fig3.tif

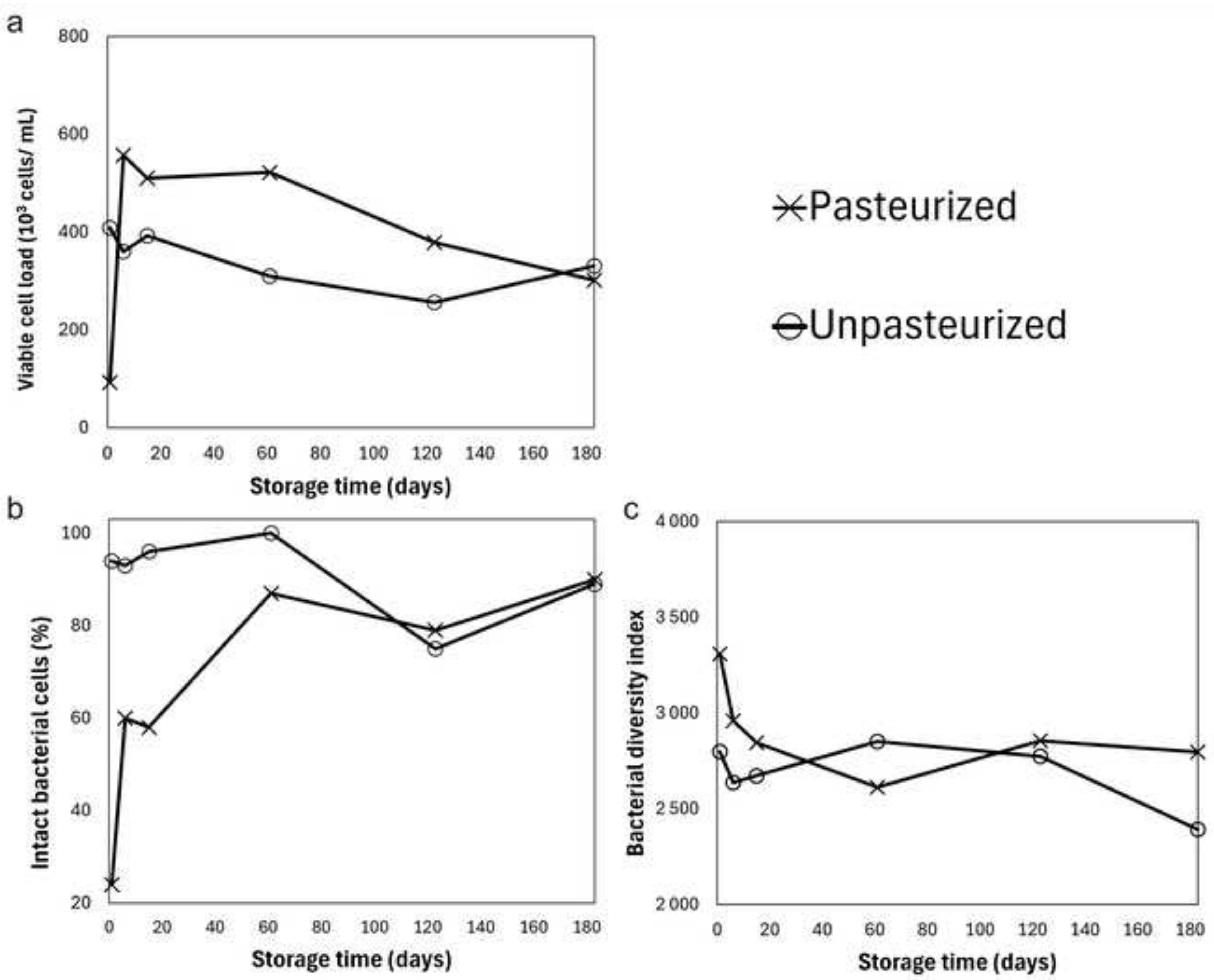

Figure 1

Click here to access/download;Figure;Fig1.tif

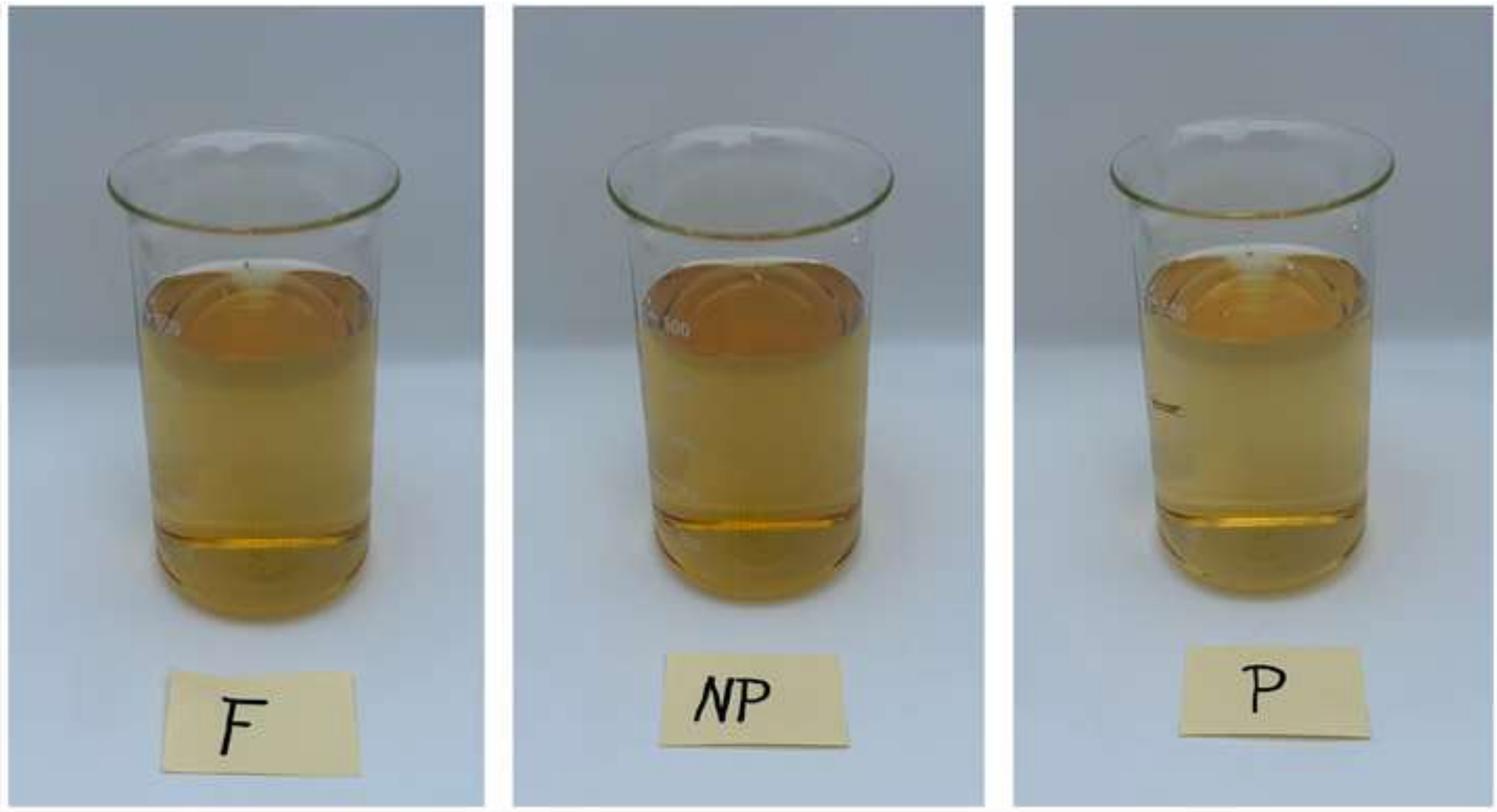

Supplement: Supplementary file 1 [file pone.0355602.s003.pdf]
