# Supplementary material for: Liquid fertilizer recovered from the effluent water of recirculating aquaculture system by reverse osmosis exhibits storage stability independent of pasteurization
Source: PLoS One. 2026 Aug 21;21(8):e0355602. doi: 10.1371/journal.pone.0355602 (PMC13496420; doi:10.1371/journal.pone.0355602)
Supplement: Supplementary file 2 [file pone.0355602.s002.docx]

The manuscript is well written and highlights an important topic related to nutrient recovery and circular bioeconomy. The findings demonstrating that RO concentrate can be stored for six months without pasteurization are valuable. However, a few improvements are required:

1. **Novelty clarification** – The introduction should more clearly explain how this study advances existing research on RO/UF-based nutrient recovery.
2. **Experimental limitations** – Only one RAS source and batch were studied; please acknowledge this limitation and suggest future validation.
3. **Statistical clarity** – Figures should include statistical significance markers (p‐values, error bars clearly labelled).
4. **Microbiological response** – The significant viable cell increase after pasteurization needs deeper explanation.
5. **Figures quality** – Improve resolution and add clearer legends and units.
