# Supplementary material for: Liquid fertilizer recovered from the effluent water of recirculating aquaculture system by reverse osmosis exhibits storage stability independent of pasteurization
Source: PLoS One. 2026 Aug 21;21(8):e0355602. doi: 10.1371/journal.pone.0355602 (PMC13496420; doi:10.1371/journal.pone.0355602)
Supplement: Supplementary file 3 [file pone.0355602.s005.docx]

**PONE-D-25-37522**

The manuscript presents valuable findings with practical implications for aquaculture and sustainable agriculture. With expanded discussion on generalizability, nutrient relevance, and microbial ecology, it will make a strong contribution to the literature.

**Minor Comments**

1. The conclusion that pasteurization is unnecessary is well supported, but the authors might expand on the economic and environmental cost-benefit analysis. Quantifying energy savings or reduced CO₂ emissions would make the argument more compelling.
2. Figures: Ensure that all figure legends are fully self-explanatory (e.g., abbreviations like EC, NC should be defined in each legend).
3. Language: Some sentences are long and could be tightened for clarity (e.g., in the introduction).
4. References: Check consistency in formatting (some entries lack full author initials or page ranges).
