# Supplementary material for: Liquid fertilizer recovered from the effluent water of recirculating aquaculture system by reverse osmosis exhibits storage stability independent of pasteurization
Source: PLoS One. 2026 Aug 21;21(8):e0355602. doi: 10.1371/journal.pone.0355602 (PMC13496420; doi:10.1371/journal.pone.0355602)
Supplement: Supplementary file 4 [file pone.0355602.s006.docx]

Response to reviewers PONE-D-25-37522. We have listed journal requirements and feedback from reviewers below, and responded to each comment in red font.

# Journal requirements:

1. Please ensure that your manuscript meets PLOS ONE's style requirements, including those for file naming.

Response: We have ensured that file names are according to PLOS ONE’s style requirements.

2. In your Methods section, provide additional information regarding the permits you obtained for the work. Please ensure you have included the full name of the authority that approved the field site access and, if no permits were required, a brief statement explaining why.

*Response: The following statement has been provided in the Materials and Methods section: Field activities were conducted at the NIBIO Landvik research station (Norwegian Institute of Bioeconomy Research, Norway).* *Access to the site and approval for experimental activities were granted by the research station manager Randi Seljåsen.*

3. Please note that PLOS One has specific guidelines on code sharing for submissions in which author-generated code underpins the findings in the manuscript. In these cases, we expect all author-generated code to be made available without restrictions upon publication of the work. Please review our guidelines at <https://journals.plos.org/plosone/s/materials-and-software-sharing#loc-sharing-code> and ensure that your code is shared in a way that follows best practice and facilitates reproducibility and reuse.

*Response: No code was generated for this study.*

4. We note that the grant information you provided in the ‘Funding Information’ and ‘Financial Disclosure’ sections do not match. When you resubmit, please ensure that you provide the correct grant numbers for the awards you received for your study in the ‘Funding Information’ section.

*Response: We have included an updated financial disclosure statement in the cover letter, as requested in the revision letter. For the “Funding information” section, no author is chosen as “grant recipient” since the grants is received by the SINTEF research institute, not the individual authors, leaving “not applicable” the best option.*

5. Thank you for stating the following in the Competing Interests/Financial Disclosure section: 
I have read the journal's policy and the authors of this manuscript have the following competing interests: Coauthor MB are employed by Columbi Farms AS who has financial interests in the technology developed through the IPN Nutricycle project. All data interpretation and research findings were conducted independently and quality assured by SINTEF to ensure compliance with PLOS one guidelines. The other authors declare no competing interests.

We note that one or more of the authors are employed by commercial companies: Columbi Farms AS and Kytos 
 
a. Please provide an amended Funding Statement declaring this commercial affiliation, as well as a statement regarding the Role of Funders in your study. If the funding organization did not play a role in the study design, data collection and analysis, decision to publish, or preparation of the manuscript and only provided financial support in the form of authors' salaries and/or research materials, please review your statements relating to the author contributions, and ensure you have specifically and accurately indicated the role(s) that these authors had in your study. You can update author roles in the Author Contributions section of the online submission form.

Please also include the following statement within your amended Funding Statement. 
“The funder provided support in the form of salaries for authors [insert relevant initials], but did not have any additional role in the study design, data collection and analysis, decision to publish, or preparation of the manuscript. The specific roles of these authors are articulated in the ‘author contributions’ section.” 
If your commercial affiliation did play a role in your study, please state and explain this role within your updated Funding Statement.

*Response: We’ve amended the Competing interests to the following, in the updated cover letter:*

*I have read the journal's policy and the authors of this manuscript have the following competing interests: Coauthor MB are employed by Columbi Farms AS who has financial interests in the technology developed through the IPN Nutricycle project. Coauthor TB is employed by Kytos BV that provide microbial community data gathered by flow cytometry, and have no financial interest in the technology developed through the IPN Nutricycle project. The Norwegian Research Council (Grant no. 336971) and the European Union's Horizon 2020 research and innovation program (Grant no. 862555) provided support in the form of grant money for the SINTEF research institute where authors DU, GR and JBS are employed and receive salary. The funders did not have any additional role in the study design, data collection and analysis, decision to publish, or preparation of the manuscript. The specific roles of these authors are articulated in the ‘author contributions’ section. All data interpretation and research findings were conducted independently and quality assured by SINTEF to ensure compliance with PLOS guidelines.*

*We’ve also amended the author roles in Author Contributions (no changes were made).*

*b. Please also provide an updated Competing Interests Statement declaring this commercial affiliation along with any other relevant declarations relating to employment, consultancy, patents, products in development, or marketed products, etc. 
 
Within your Competing Interests Statement, please confirm that this commercial affiliation does not alter your adherence to all PLOS ONE policies on sharing data and materials by including the following statement: "This does not alter our adherence to PLOS ONE policies on sharing data and materials.” (as detailed online in our guide for authors* [*http://journals.plos.org/plosone/s/competing-interests*](http://journals.plos.org/plosone/s/competing-interests)*) . If this adherence statement is not accurate and there are restrictions on sharing of data and/or materials, please state these. Please note that we cannot proceed with consideration of your article until this information has been declared. 
 
Please include both an updated Funding Statement and Competing Interests Statement in your cover letter. We will change the online submission form on your behalf.*

*Response: We have included an updated financial disclosure statement and updated competing interest section in the cover letter as requested.*

*6. When completing the data availability statement of the submission form, you indicated that you will make your data available on acceptance. We strongly recommend all authors decide on a data sharing plan before acceptance, as the process can be lengthy and hold up publication timelines. Please note that, though access restrictions are acceptable now, your entire data will need to be made freely accessible if your manuscript is accepted for publication. This policy applies to all data except where public deposition would breach compliance with the protocol approved by your research ethics board. If you are unable to adhere to our open data policy, please kindly revise your statement to explain your reasoning and we will seek the editor's input on an exemption. Please be assured that, once you have provided your new statement, the assessment of your exemption will not hold up the peer review process.*

*Response: All data used in this study have been deposited in the Open Science Framework (OSF) and are publicly available, as described and referenced in section 2.4 Statistics.*

*7. If the reviewer comments include a recommendation to cite specific previously published works, please review and evaluate these publications to determine whether they are relevant and should be cited. There is no requirement to cite these works unless the editor has indicated otherwise.*

*Response: The reviewers did not include recommendations to cite specific works.*

*8. Please review your reference list to ensure that it is complete and correct. If you have cited papers that have been retracted, please include the rationale for doing so in the manuscript text, or remove these references and replace them with relevant current references. Any changes to the reference list should be mentioned in the rebuttal letter that accompanies your revised manuscript. If you need to cite a retracted article, indicate the article’s retracted status in the References list and also include a citation and full reference for the retraction notice.*

*Response: We have reviewed the reference list and have made no changes.*

# Reviewer 1

*The manuscript is well written and highlights an important topic related to nutrient recovery and circular bioeconomy. The findings demonstrating that RO concentrate can be stored for six months without pasteurization are valuable. However, a few improvements are required:*

*Novelty clarification – The introduction should more clearly explain how this study advances existing research on RO/UF-based nutrient recovery.*

*Response: Thank you for the positive feedback and suggestions for improvements. We have edited the introduction to include how our study advances existing research on RO/UF-based nutrient recovery.
 
Experimental limitations – Only one RAS source and batch were studied; please acknowledge this limitation and suggest future validation.*

*Response: We have acknowledged this limitation in the Conclusion.
 
Statistical clarity – Figures should include statistical significance markers (p‐values, error bars clearly labelled).*

Thank you for the feedback. The data presenting chemical composition includes error bars clearly labelled, and we have chosen to present statistically significant difference between relevant sample points the text while discussing results. In the case of data derived by flow cytometry, single samples were analysed as described in Prest et al (2013), <https://doi.org/10.1016/j.watres.2013.07.051>, which documents the extremely low measurement variability when using this method mainly due to the statistical replication inherent in a single sample containing millions of individually counted cells. Error bars are therefore not applicable.

*Microbiological response – The significant viable cell increase after pasteurization needs deeper explanation.*

*Response: We have expanded section 3.1 to provide a deeper explanation of this finding. While this represents an interesting observation, the establishment of a stable viable cell load over time independent of treatment is (in the context of storage stability) the key message we wish to communicate.
 
Figures quality – Improve resolution and add clearer legends and units.*

*Response: We have ensured that figures uploaded to the journal are within the quality requirement of 300-600 dpi resolution.*

# Reviewer 2

*Your manuscript could be better if you compare your results with those main crops nutrient needs in Norway. Is interesting your foundings but it lacks of the support of the corps needs and more of what farmers need to improve their corps. This comparation will improve your work and give you more bases to have a more complete discussion obout your results. For now, your results are the preliminary part of what you really want to demonstrate with your research.*

*Response: Thank you for your feedback. You’re right, this study represents a small step towards the utilization of organic fertilizers derived from RAS as an alternative to conventional fertilizers, and there’s still many unexplored research areas. However, expanding the study to include crop nutrient needs falls outside of the study’s scope, which is storage stability. We do, however, acknowledge that the user aspect is an important next step in the development of RO-concentrate as an alternative to mineral fertilizer. We have therefore included a short discussion in the Conclusion on the main characteristics of the RO concentrate as a fertilizer, how it can be enriched or balanced to meet the nutrient requirements of different crops, and what this implies for future research.*

Reviewer 3

*This is a valuable article in sustainable Aquacultur and Agriculture activities. Minor revisions are recommended. 
I suggest to create a table for summerizing the chemical and microbial changes throughout the experimental period for both the Treated and the control Nutrient Concentrate.*

*Response: Thank you for your feedback. This data is already displayed as time-series plots in the manuscript, and the table you suggest is already available in the raw data supplied in the Open Science Framework referenced in section 2.4 (Statistics). In our opinion, data should not be presented in both figures and tables, if the figure already allows the reader to extract the relevant values. In this case, we believe a graphical representation using figures is more suitable to display trends over time.*
