# Supplementary material for: Liquid fertilizer recovered from the effluent water of recirculating aquaculture system by reverse osmosis exhibits storage stability independent of pasteurization
Source: PLoS One. 2026 Aug 21;21(8):e0355602. doi: 10.1371/journal.pone.0355602 (PMC13496420; doi:10.1371/journal.pone.0355602)
Supplement: Supplementary file 5 [file pone.0355602.s007.docx]

Response to Reviewers

Dear Editor, please find below our responses to the reviewers’ comments. The accompanying file, *Revised Manuscript with Track Changes*, highlights all modifications made in response to each comment.

**PONE-D-25-37522**

*The manuscript presents valuable findings with practical implications for aquaculture and sustainable agriculture. With expanded discussion on generalizability, nutrient relevance, and microbial ecology, it will make a strong contribution to the literature.*

***Minor Comments***

1. *The conclusion that pasteurization is unnecessary is well supported, but the authors might expand on the economic and environmental cost-benefit analysis. Quantifying energy savings or reduced CO₂ emissions would make the argument more compelling.*

Thank you for this constructive suggestion. We agree that an environmental and economic assessment of pasteurization could further strengthen the discussion. We explored how such an analysis might be incorporated, however, performing a meaningful and methodologically sound assessment would require developing detailed assumptions about a full‑scale pasteurization system, including heating infrastructure, pumps, heat exchangers, and operational parameters. Because energy use and associated CO₂ emissions depend strongly on the specific equipment design and process configuration, a theoretical calculation based solely on the energy required to heat water using the formula $Q=m\cdot c\cdot\Delta T$ would not reflect real industrial performance and could be misleading.

A comprehensive cost–benefit or life‑cycle assessment would therefore necessitate an entirely new methodological section, including system boundaries, technology specifications, and sensitivity analyses. As this lies outside the scope of our current research, which focuses on the microbiological and chemical changes in pasturized and unpasteurized concentrate, we chose not to include an environmental footprint analysis in this manuscript.

2. *Figures: Ensure that all figure legends are fully self-explanatory (e.g., abbreviations like EC, NC should be defined in each legend).*

We have checked and confirm that all figure legends are self-explanatory. Figure 1 use the abbreviations “newly produced (F)”, “not pasteurized (NP)” and “pasteurized (P)”, Figure 2 use “EC = Electrical conductivity”; “NO3 = Nitrate”; “K = Potassium”; “Fe = Iron”, and figure 3 does not use abbreviations. All these abbreviations were already included in the figure legends of the submitted manuscript, so no changes has been made.

3. *Language: Some sentences are long and could be tightened for clarity (e.g., in the introduction).*

Thank you for the helpful feedback on improving the clarity of the manuscript. We have revised the text accordingly, with particular focus on shortening and clarifying sentences in the introduction. The updates are available in the file titled “Revised manuscript with track changes.”

4. *References: Check consistency in formatting (some entries lack full author initials or page ranges).*

Thank you for the feedback, we have gone through the sources formatting to ensure correct formatting of author initials and page ranges.

5. *In addition, please add Data Availability Statement, placed before the Acknowledgment --> “The data that support the findings of this study are openly available in OSF repository at* [*https://osf.io/9bqpc/?view_only=b61606c3dd524ce3a9a3692b7dd7c81*](https://osf.io/9bqpc/?view_only=b61606c3dd524ce3a9a3692b7dd7c81)*”*

Thank you for the clear and concise feedback, we have now included the Data Availability Statement with the suggested text in the revised manuscript.

6. *Reviewer #1: The manuscript presents valuable findings with practical implications for aquaculture and sustainable agriculture.*
7. *Reviewer #2: The arguments in their results are very weak and require further review of the literature. Most of your assumptions lack of technical and literature support in each area of their results. I recommend conducting a more in-depth literary review to support your arguments and the evidence you've found in your results.*

Thank you for reviewing our manuscript. It is unclear to us which arguments you are referring to specifically, so we have systematically gone through the results and discussion section and made the following changes: In Subchapter 3.1, where we discuss the effect of pasteurisation on pH, EC, and concentration of dissolved ions, we have changed the text to clarify our thought process and hypothesis development, and added references that support our observations. We have also altered the discussion on the observed drop in pH in the unpasteurised samples towards the end of the storage period. In subchapter 3.2, we have made slight changes throughout the text to clarify our arguments and added references to support our arguments.
