# Supplementary material for: Liquid fertilizer recovered from the effluent water of recirculating aquaculture system by reverse osmosis exhibits storage stability independent of pasteurization
Source: PLoS One. 2026 Aug 21;21(8):e0355602. doi: 10.1371/journal.pone.0355602 (PMC13496420; doi:10.1371/journal.pone.0355602)
Supplement: Supplementary file 6 [file pone.0355602.s008.docx]

**Response to Reviewers**

Dear Editor, please find below our responses to the reviewers’ comments. The accompanying file, Revised Manuscript with Track Changes, highlights all modifications made in response to each comment.

***From Academic Editor:***

*Please download the attachment from Reviewer 1. There are also still many issues to clarify per Reviewer 1's suggestions. In addition, the data shown in the manuscript are two figures (with one with 5 panels, and the second is just qualitative/visual image). I agree with Reviewer 1 who requires new data (and discussions), with the objective to have a solid manuscript.*

Dear Editor,

Thank you for your feedback. We have addressed the comments from Reviewer 1 below.

With regard to the data presented in the Results and Discussion section, we would like to respectfully clarify that the manuscript includes more quantitative data than what is implied in your comment.

Specifically, Table 1 presents the initial composition of the nutrient concentrate, Figure 2 presents the change of chemical parameters during storage, and Figure 3 presents the change of microbiological parameters during storage. Of these, you only include figure 2 in your comment. In addition, the complete chemical composition and additional microbiological parameters are provided in the Supplementary Materials available at <https://osf.io/9bqpc/overview?view_only=b61606c3dd524ce3a9a3692b7dd7c81e>. As you point out figure 1 is only a visual image, which we have included in the article to provide context for the reader.

We therefore argue that the manuscript contains sufficient data for publication.

***Reviewers' comments:*** *Reviewer's Responses to Questions*

***Comments to the Author*** *1. If the authors have adequately addressed your comments raised in a previous round of review and you feel that this manuscript is now acceptable for publication, you may indicate that here to bypass the “Comments to the Author” section, enter your conflict of interest statement in the “Confidential to Editor” section, and submit your "Accept" recommendation.*

*Reviewer #1: All comments have been addressed*

*Reviewer #2: All comments have been addressed*

*2. Is the manuscript technically sound, and do the data support the conclusions?

The manuscript must describe a technically sound piece of scientific research with data that supports the conclusions. Experiments must have been conducted rigorously, with appropriate controls, replication, and sample sizes. The conclusions must be drawn appropriately based on the data presented.*

*Reviewer #1: Yes*

*Reviewer #2: Yes*

*3. Has the statistical analysis been performed appropriately and rigorously?*

*Reviewer #1: Yes*

*Reviewer #2: Yes*

*4. Have the authors made all data underlying the findings in their manuscript fully available?
Reviewer #1: Yes*

*Reviewer #2: Yes*

*5. Is the manuscript presented in an intelligible fashion and written in standard English?

PLOS ONE does not copyedit accepted manuscripts, so the language in submitted articles must be clear, correct, and unambiguous. Any typographical or grammatical errors should be corrected at revision, so please note any specific errors here.*

*Reviewer #1: Yes*

*Reviewer #2: Yes*

*6. Review Comments to the Author

Please use the space provided to explain your answers to the questions above. You may also include additional comments for the author, including concerns about dual publication, research ethics, or publication ethics. (Please upload your review as an attachment if it exceeds 20,000 characters)*

*Reviewer #1: Major concerns:
• Experimental design is limited (single source, single batch; microbiology n=1), reducing reliability.*

The inorganic concentrate used in this study was produced in a unique reverse osmosis pilot system permanently connected to an experimental RAS facility owned by NIBIO Landvik. Including multiple sources of RAS effluent water was therefore not practically feasible. In the case of data derived by flow cytometry, single samples were analysed as described in Prest et al (2013), <https://doi.org/10.1016/j.watres.2013.07.051>, which documents the extremely low measurement variability when using this method mainly due to the statistical replication inherent in a single sample containing millions of individually counted cells. While we believe this study is a valuable contribution to the literature in its current form, we have acknowledged the limitations highlighted in this review in our conclusion, which includes the following statement:

“As this study relied on a single source of RAS wastewater, future studies should include multiple sources of RAS wastewater and different batches of RO concentrate to further validate the findings.”

*• Statistical analysis is inadequate; time-series data require more robust methods.*

As stated in the previous comment, we acknowledge the limitations of the study in the conclusion with the statement “As this study relied on a single source of RAS wastewater, future studies should include multiple sources of RAS wastewater and different batches of RO concentrate to further validate the findings.” Otherwise, appropriate statistical methods were used to analyse the results, given the experimental design. We would also like to point out that both reviewers have answered “yes” to the question of whether statistical analysis been performed appropriately and rigorously above.

*• Claims of “microbial stability” are somewhat overstated given observed fluctuations.*

We argue that the system is microbiologically stable because we do not observe exponential growth or large fluctuations over time as one would expect in a system dominated by microorganisms if conditions favoured growth. The observed fluctuations are limited to the samples that were pasteurised, where an initial reduction in the viable cell load and intact bacterial cells quickly recovered to a level which remained stable during the storage period independent of treatment.

*• Mechanisms behind nutrient loss after pasteurization are speculative and need validation.*

We agree that the mechanisms behind nutrient loss after pasteurization need validation in future studies that are designed to investigate these observations. However, the scope of this study was to investigate the storage stability of an inorganic concentrate produced from RAS effluent water with and without pasteurization. It is, to our knowledge, the first time this specific concept has been studied, and is an important contribution towards the development of viable alternatives to conventional mineral fertilizers.

*• No agronomic validation (e.g., plant trials) to support fertilizer applicability.*

This study was designed to investigate the storage stability of an inorganic concentrate produced from RAS effluent water with and without pasteurization. Agronomic validation of the fertilizer applicability will be evaluated in future plant trials, as we have recommended in the conclusion section of the manuscript.

*Minor concerns:
• Improve clarity, reduce repetition, and refine language.*

We have edited the manuscript to improve clarity and reduce repetition. We would also like to point out that both reviewers have answered “yes” to the question of whether the manuscript is presented in an intelligible fashion above.

*• Correct duplicated figure captions and improve figure presentation.*

We have ensured consistent figure captions and references throughout the text.

*• Ensure consistency in units and terminology (avoid labeling as purely “organic fertilizer”).*

We have reviewed and altered the labelling, units and terminology throughout the manuscript.
