# Supplementary material for: Liquid fertilizer recovered from the effluent water of recirculating aquaculture system by reverse osmosis exhibits storage stability independent of pasteurization
Source: PLoS One. 2026 Aug 21;21(8):e0355602. doi: 10.1371/journal.pone.0355602 (PMC13496420; doi:10.1371/journal.pone.0355602)
Supplement: Supplementary file 7 [file pone.0355602.s009.docx]

**Academic Editor's note:**

**The situation of split decision for this manuscript is quite tough. Therefore, please provide a highly detailed point-by-point response showing how each individual question from each reviewer is addressed, and where is the location of the response in the revised manuscript. This is crucial in order to show how this submission can contribute significantly to the development of science and technology.**

*Dear Editor,*

*Thank you for evaluating our manuscript and for considering it for publication in PLOS ONE.*

*Below, we provide a detailed, point-by-point response to each reviewer comment and indicate the corresponding line numbers in the revised manuscript where changes have been made as requested. Please note that the line numbers refer to the revised manuscript file entitled* ***"****Manuscript****"*** *and not to the file* ***"****Revised Manuscript with Track Changes."*

*As this is the fourth round of review for this manuscript in PLOS ONE, following a review process that has extended 12 months, we hope that the editorial team will be able to reach a final decision during the current review cycle.*

*We appreciate the time and effort invested by the editor and reviewers in evaluating our work.*

**Reviewer #1: all comments given are done appropriately. this is important paper in advanced aquaculture field where RAS technique is one of that.**

**Reviewer #2: (No Response)**

**Reviewer #4: This work recovered plant nutrients from salmon RAS effluent using a RO system and studied the storage stability of the resulting RO concentrate over six months. It is concluded that RAS NC is stable when stored at room temperature without light for six months, regardless of pasteurization. Some more comments are:

1. Flocculation, UF and RO are used to treat the RAS effluent. More discussion and data on the system should be provided. For example, what are the size, rejection, recovery of the UF and RO units?**

*Dear reviewer, thank you for the comment.*

*The requested details about operation parameters of the RO and UF system are unfortunately not available due to confidentiality. While we agree the information would provide context, we argue that it is not essential as the study don’t aim to optimize the UF/RO process, nor to compare different concentrations technologies. The relevant information to study the shelf life of a concentrate is the initial composition of the concentrate as well as changes in composition during storage, which is clearly presented and discussed throughout the article.*

*Therefore, while additional operational details of the UF and RO system would be informative, we do not consider their absence to affect the interpretation of the results or the conclusions drawn regarding concentrate shelf life.*

**What are the requirements for plant nutrients in terms of composition and concentration? How is RO concentrate compared to the requirements?**

*Different cultivation systems require different fertilizer compositions, and a comparison is therefore dependent on the use case. The concentrate produced in our study, as now stated in the manuscript, is a nitrogen-rich concentrate and could be used accordingly or balanced by adding individual macronutrients to meet the nutrient requirements of specific crops. For example, phosphorous must be added to the concentrate to create a classical NPK-fertilizer, alternatively, the concentrate is suitable as a nitrogen-rich fertilizer in areas where the use of phosphorous is discouraged or prohibited due to high soil phosphorous.*

*In response to this comment, we have expanded upon the discussion in line 342-349 of the revised manuscript.*

**2. Why is pasteurization (heat treatment) carried out at 80 oC for 10 minutes? Is this an optimum condition?**

*We chose to use heat treatment at 80 °C for 10 minutes because it is expected to effectively eliminate vegetative cells based on published D_T_-values (*[*https://doi.org/10.3390/foods6120107*](https://doi.org/10.3390/foods6120107)*). We avoided thermal sterilization at autoclave conditions because 1) we wanted to minimize precipitation of minerals in our concentrate, while still achieving thermal pasteurization, and 2) in an industrial setting, a moderate thermal treatment such as the one applied here is more economically viable than sterilization. The treatment provided a baseline for continued development, and if our results had indicated quality degradation during storage due to growth of microorganisms, a continued research focus would have targeted optimizing a thermal treatment to achieve sufficient storage stability of the concentrate. The revised manuscript has been updated with the source in line 135 – 136.*

3. Visual assessment of physical characteristics revealed no change in precipitation. Why is precipitation highlighted as the reason for reduced Fe concentration?

*The visual assessment cannot exclude precipitation as a possibility due to the methodology, where the non-transparent storage containers were thoroughly mixed before being poured into the sample glass for analysis. Such mixing could have resuspended any precipitates, preventing its visual detection. We would also highlight that our hypothesis is conjectured and requires further study. To clarify this, we have made the following changes in the revised manuscript:
Line 141-142: Added the specific methodology on sampling for visual characteristics.*

*Line 217-225: Added discussion on precipitation as a theory explaining the observed changes, and discussion on why precipitation was not observed visually, if it was there. Underscores that our hypothesis is conjectured and requires further study.*

**4. It is hypothesized that the observed increase in the solution’s pH has resulted in
precipitation of iron and other dissolved ions. This claim needs to be supported with calculation. The pH is increased from 6.97 to 7.37. How much precipitation can happen with this change of pH?**

*Dear reviewer, thank you for the comment.*

*We have edited the hypothesis explaining the observed changes in pH, EC and dissolved ions during pasteurization and storage in line 217-225 and 229-230 in the revised manuscript. The explanation now includes microbial activity in addition to precipitation and underscores that theory is conjectured and requires further study. We agree that a calculation would be appropriate if iron was the only ion in the solution and we knew precipitation was occurring. However, the solution represents a complex system containing a variety of ions, microbiological organisms, and potentially other compounds that may contribute to precipitation and affect EC. Given these complexities, a quantitative estimation of precipitation based on pH change alone would be difficult to justify and has therefore not been included in the manuscript.*

**5. With heat treatment, why is the pH increased from 6.97 to 7.37?**

*We speculate that the observed difference in pH was caused by the thermal treatment, which may cause small pH-changes due to loss of dissolved CO_2_ and precipitation of hydrolysing metals.*

**6. It is said that EC indicates temporal fluctuations in the degree of mineral precipitation during the storage period. Why are there temporal fluctuations?**

*Dear reviewer, thank you for the feedback.*

*We observed temporal fluctuations in the EC and in elements dissolved in the solution during the storage period. One theory to explain the observation is mineral precipitation due to changes in pH. We have expanded upon the discussion and underscored that this hypothesis is conjectured and requires further study, in line 217-225 of the revised manuscript. Another possible explanation for the observed temporal fluctuations is microbiological activity, which we have now included in line 229-231and later in the revised manuscript. We have revised the section to describe the observed temporal fluctuation and explore potential mechanisms underlying this behaviour, rather than attributing it directly to precipitation.*

**7. The viable cell load increased slightly in the unpasteurized control towards the end of the storage period, which may indicate the onset of spoilage. Do this mean the pasteurized sample have longer storage stability?**

*Possibly. Currently, the manuscript highlights this observation and comments how this may indicate the onset of spoilage in the unpasteurized control in line 325-327 of the revised manuscript. However, we do not have data to support further comments on the stability of the samples beyond 6 months storage, which is why we recommend longer storage studies to be conducted as further research.*

**8. UF is effective in removing microorganisms. Can you use UF to pasteurize the sample, in steady of 80 oC for 10 minutes?**

*This is also our hypothesis as stated in the manuscript; in similarity to pasteurization, ultrafiltration removes vegetative cells, which has likely influenced (increased) the storage stability of the control sample and may, if substantiated, replace pasteurisation as a conservative treatment.*

**Reviewer #5: The author needs to address these to improve their study:**

**1. There is substantial research on using RO after effluent treatment, with a strong focus on the resulting concentrate. Pasteurization has also been used to improve the stability of the effluent. So, what is actually the novelty of this work compared to those reported works? What is the main scientific contribution of recovering liquid fertilizer from RAS effluent using reverse osmosis?**

*We agree that the literature on effluent concentrate production by RO is substantial. The novelty of this work, and the main scientific contribution, is not in the production technology itself or the composition of the concentrate, but rather the investigation of long-term storage stability of RAS effluent concentrate produced by reverse osmosis as a practical barrier for large-scale utilisation, which is poorly documented in the literature. The data this study provides contributes towards the use of industrial effluent concentrates as fertilizers in conventional agriculture, where long-term storage stability is a key parameter. We would also like to use this opportunity to provide a quote from Plos one’s website: “The editors make decisions on submissions based on scientific rigor, regardless of novelty.”*

**2. I also did not find why RO is selected over other nutrient concentration technologies, such as nanofiltration, forward osmosis, membrane distillation, evaporation, or adsorption. There is no systematic approach to selecting RO in their work.**

*The aim of the current study was to investigate the storage stability of a nutrient concentrate produced by RO with and without pasteurization, not to compare different nutrient concentration technologies. Therefore, a detailed description on the advantages and disadvantages of RO compared to other technologies are not included in the article. As a response to this comment, we have however added a sentence in line 100-102 in the introduction section of the article that briefly explain why RO was used in the current study.*

*Prior to building the reverse osmosis pilot system the project group did perform a structured technology review to find a suitable technology to our purpose, where reverse osmosis and nanofiltration emerged as the most suitable options. We chose RO due to the technology’s maturity and extensive application in existing water treatment infrastructure. Meanwhile, other alternatives such as evaporation would likely be to energy demanding to be feasible at larger scales.*

**3. What about RAS composition before RO treatment? I found only RAS NC composition and characteristics. They need to compare the composition before and after RO treatment.**

*Dear reviewer, thank you for the comment.*

*The requested details about RAS water composition before treatment with RO are unfortunately not available due to confidentiality. While we agree the information would provide context, we argue that it is not essential. The relevant information for studying the shelf life of a concentrate is the initial composition of the concentrate as well as changes in composition during storage, which is clearly presented and discussed throughout the article.*

*Therefore, while additional details on the RAS water composition would be informative, we do not consider their absence to affect the interpretation of the results or the conclusions drawn regarding concentrate shelf life.*

**4. Figure 1 shows physical characteristics. I was thinking it was something like density and other physical properties. But it is basically only the image of the RAS. This is not physical characteristics. It is just physical appearance. So better to use another caption to avoid misleading about something else.**

*Thank you for the feedback. We agree that the original figure caption was misleading and have therefore revised it to “visual characteristics” in the figure caption and “Visual assessment” in line 188, as suggested. Figure 1 is included to provide visual context for the reader, while data on composition and stability are presented in the subsequent tables and figures.*

5. Does the recovered RO concentrate meet basic requirements for use as a liquid fertilizer? I mean, let’s say by comparing the recovered fertilizer with commercial liquid fertilizers?

*Different cultivation systems require different fertilizer compositions, and a comparison is therefore dependent on the use case. The concentrate produced in our study, as stated in the manuscript, is a nitrogen-rich concentrate and can be used accordingly, or balanced by adding individual macronutrients to meet the nutrient requirements of specific crops. For example, phosphorous must be added to the concentrate to create a classical NPK-fertilizer, alternatively, the concentrate is suitable as a nitrogen-rich fertilizer in areas where the use of phosphorous is discouraged or prohibited due to high soil phosphorous.*

*In response to this comment, we have expanded upon the discussion given in the original manuscript, in line 342 – 349.*

6. Under the tested dark-storage conditions, pasteurization did not significantly affect the measured microbial load, intact bacterial cell concentration, or bacterial diversity of the RO-concentrated RAS liquid fertilizer. But it is not sufficient on its own to support a broad practical or commercial claim that pasteurization is unnecessary or obsolete as they concluded. They should test specifically for safety-relevant microorganisms, not only total viable cells to provide strong claim on

*Thank you for the feedback. We agree that the current study is not sufficient to support broad practical or commercial claims, nor has it been our intention to make such claims. We have made the following changes in the revised manuscript to acknowledge the limitations of the study and moderate our claims.*

*Line 244 – 249 specify that the discussion and results concern the specific batch that was studied.*

*Line 325 – 329 and line 332 – 334 in the conclusion is edited to clearly acknowledge limitations and moderate claims on storage stability.*

*In addition, we would like to point out the following unchanged sections in the manuscript that also acknowledge the studies limitations:
Line 295-300 in the revised manuscript clearly address the increase in microbiological activity towards the end of the storage study (no changes made)
Line 334-336 in the revised manuscript acknowledge the limitations by having tested only one batch of RAS water, and that longer storage periods should be tested to determine the final shelf life (no changes made).*

*Finally, we acknowledge that safety-relevant microorganisms are important for the commercial applicability of the concentrate as a fertilizer, although we would argue that expanding the focus to include product safety parameters goes beyond the scope of the study, which is limited to the stability of the concentrate in terms of plant nutrient composition.*

7. Could the process support circular economy principles in aquaculture-agriculture integration?

*Definitely, and this is of course the original motivation for performing this study, as stated in the manuscript. We also feel that it is important to acknowledge that the scale of aquaculture (and therefore the available nutrient concentrate volumes) is so large that the solutions and technologies developed, and recipients, must be scaled accordingly.*

**Reviewer #6:**

**1. Please add a flowchart to show more clearly the experimental process.**

*Thank you for the feedback. As response to this comment we have added a graphical abstract presenting a flowchart of the experimental process.*

**2. The conclusion that the concentrate is “microbiologically stable” may be somewhat overstated considering the observed increase in viable cell load and reduction in bacterial diversity toward the end of the storage period in the unpasteurized samples. These trends may indicate the early onset of spoilage, and the discussion should acknowledge this limitation more cautiously.**

*Thank you for the feedback. We agree that the current study is not sufficient to support broad practical or commercial claims, and that the current conclusion is somewhat overstated. We have made the following changes in the manuscript to clearly acknowledge the limitations of the study and moderate our claims.*

*Line 244 – 249 specify that the discussion and results concern the specific batch that was studied.*

*Line 325 – 329 and line 332 – 334 in the conclusion is edited to clearly acknowledge limitations and moderate claims on storage stability.*

*In addition, we would like to point out the following unchanged sections in the manuscript that also acknowledge the studies limitations:
Line 295-300 in the revised manuscript clearly address the increase in microbiological activity towards the end of the storage study (no changes made)
Line 334-336 in the revised manuscript acknowledge the limitations by having tested only one batch of RAS water, and that longer storage periods should be tested to determine the final shelf life (no changes made).*

**3. Although the study demonstrates storage stability, the practical fertilizer applicability of the recovered concentrate is not experimentally validated. No plant growth trials, nutrient uptake studies, or agronomic performance assessments were conducted to confirm the effectiveness of the product as an alternative fertilizer source. Including preliminary cultivation experiments would substantially enhance the practical significance of the manuscript.**

*We agree that cultivation trials are necessary to validate the concentrate as a fertilizer, which is why we have expanded upon the discussion of the concentrate as a fertilizer and recommend plant cultivation trials to be conducted in further studies on line 242 -349 in the revised manuscript. The aim of the current study was to assess the storage stability of the concentrate, not to test its performance as a fertilizer. Therefore, plant cultivation trials are outside the scope of this study.*
