# Supplementary material for: Liquid fertilizer recovered from the effluent water of recirculating aquaculture system by reverse osmosis exhibits storage stability independent of pasteurization
Source: PLoS One. 2026 Aug 21;21(8):e0355602. doi: 10.1371/journal.pone.0355602 (PMC13496420; doi:10.1371/journal.pone.0355602)
Supplement: Peer review history

# Peer review: Decision Letter 0

**DOI:** 10.1371/journal.pone.0355602.r001

**Authors:** Susmita Lahiri (Ganguly)

16 Dec 2025

Dear Dr. Ulleberg,

Thank you for submitting your manuscript to PLOS ONE. After careful consideration, we feel that it has merit but does not fully meet PLOS ONE’s publication criteria as it currently stands. Therefore, we invite you to submit a revised version of the manuscript that addresses the points raised during the review process.

Please submit your revised manuscript by Jan 30 2026 11:59PM. If you will need more time than this to complete your revisions, please reply to this message or contact the journal office at plosone@plos.org. When you're ready to submit your revision, log on to https://www.editorialmanager.com/pone/ and select the 'Submissions Needing Revision' folder to locate your manuscript file.

- A letter that responds to each point raised by the academic editor and reviewer(s). You should upload this letter as a separate file labeled 'Response to Reviewers'.
- A marked-up copy of your manuscript that highlights changes made to the original version. You should upload this as a separate file labeled 'Revised Manuscript with Track Changes'.
- An unmarked version of your revised paper without tracked changes. You should upload this as a separate file labeled 'Manuscript'.

If applicable, we recommend that you deposit your laboratory protocols in protocols.io to enhance the reproducibility of your results. Protocols.io assigns your protocol its own identifier (DOI) so that it can be cited independently in the future. For instructions see: https://journals.plos.org/plosone/s/submission-guidelines#loc-laboratory-protocols. Additionally, PLOS ONE offers an option for publishing peer-reviewed Lab Protocol articles, which describe protocols hosted on protocols.io. Read more information on sharing protocols at https://plos.org/protocols?utm_medium=editorial-email&utm_source=authorletters&utm_campaign=protocols.

We look forward to receiving your revised manuscript.

Kind regards,

Susmita Lahiri (Ganguly)

Academic Editor

PLOS One

Journal Requirements:

When submitting your revision, we need you to address these additional requirements.

1. Please ensure that your manuscript meets PLOS ONE's style requirements, including those for file naming. The PLOS ONE style templates can be found at

https://journals.plos.org/plosone/s/file?id=wjVg/PLOSOne_formatting_sample_main_body.pdf and

https://journals.plos.org/plosone/s/file?id=ba62/PLOSOne_formatting_sample_title_authors_affiliations.pdf

2. In your Methods section, please provide additional information regarding the permits you obtained for the work. Please ensure you have included the full name of the authority that approved the field site access and, if no permits were required, a brief statement explaining why.

3. Please note that PLOS One has specific guidelines on code sharing for submissions in which author-generated code underpins the findings in the manuscript. In these cases, we expect all author-generated code to be made available without restrictions upon publication of the work. Please review our guidelines at https://journals.plos.org/plosone/s/materials-and-software-sharing#loc-sharing-code and ensure that your code is shared in a way that follows best practice and facilitates reproducibility and reuse.

4. We note that the grant information you provided in the ‘Funding Information’ and ‘Financial Disclosure’ sections do not match.

When you resubmit, please ensure that you provide the correct grant numbers for the awards you received for your study in the ‘Funding Information’ section.

5. Thank you for stating the following in the Competing Interests/Financial Disclosure section:

I have read the journal's policy and the authors of this manuscript have the following competing interests: Coauthor MB are employed by Columbi Farms AS who has financial interests in the technology developed through the IPN Nutricycle project. All data interpretation and research findings were conducted independently and quality assured by SINTEF to ensure compliance with PLOS one guidelines. The other authors declare no competing interests.

We note that one or more of the authors are employed by commercial companies: Columbi Farms AS and Kytos

a. Please provide an amended Funding Statement declaring this commercial affiliation, as well as a statement regarding the Role of Funders in your study. If the funding organization did not play a role in the study design, data collection and analysis, decision to publish, or preparation of the manuscript and only provided financial support in the form of authors' salaries and/or research materials, please review your statements relating to the author contributions, and ensure you have specifically and accurately indicated the role(s) that these authors had in your study. You can update author roles in the Author Contributions section of the online submission form.

Please also include the following statement within your amended Funding Statement.

“The funder provided support in the form of salaries for authors [insert relevant initials], but did not have any additional role in the study design, data collection and analysis, decision to publish, or preparation of the manuscript. The specific roles of these authors are articulated in the ‘author contributions’ section.”

If your commercial affiliation did play a role in your study, please state and explain this role within your updated Funding Statement.

b. Please also provide an updated Competing Interests Statement declaring this commercial affiliation along with any other relevant declarations relating to employment, consultancy, patents, products in development, or marketed products, etc.

Within your Competing Interests Statement, please confirm that this commercial affiliation does not alter your adherence to all PLOS ONE policies on sharing data and materials by including the following statement: "This does not alter our adherence to PLOS ONE policies on sharing data and materials.” (as detailed online in our guide for authors http://journals.plos.org/plosone/s/competing-interests) . If this adherence statement is not accurate and there are restrictions on sharing of data and/or materials, please state these. Please note that we cannot proceed with consideration of your article until this information has been declared.

Please include both an updated Funding Statement and Competing Interests Statement in your cover letter. We will change the online submission form on your behalf.

6. When completing the data availability statement of the submission form, you indicated that you will make your data available on acceptance. We strongly recommend all authors decide on a data sharing plan before acceptance, as the process can be lengthy and hold up publication timelines. Please note that, though access restrictions are acceptable now, your entire data will need to be made freely accessible if your manuscript is accepted for publication. This policy applies to all data except where public deposition would breach compliance with the protocol approved by your research ethics board. If you are unable to adhere to our open data policy, please kindly revise your statement to explain your reasoning and we will seek the editor's input on an exemption. Please be assured that, once you have provided your new statement, the assessment of your exemption will not hold up the peer review process.

7. If the reviewer comments include a recommendation to cite specific previously published works, please review and evaluate these publications to determine whether they are relevant and should be cited. There is no requirement to cite these works unless the editor has indicated otherwise.

8. Please review your reference list to ensure that it is complete and correct. If you have cited papers that have been retracted, please include the rationale for doing so in the manuscript text, or remove these references and replace them with relevant current references. Any changes to the reference list should be mentioned in the rebuttal letter that accompanies your revised manuscript. If you need to cite a retracted article, indicate the article’s retracted status in the References list and also include a citation and full reference for the retraction notice.

[Note: HTML markup is below. Please do not edit.]

Reviewers' comments:

Reviewer's Responses to Questions

**Comments to the Author**

1. Is the manuscript technically sound, and do the data support the conclusions?

Reviewer #1: Yes

Reviewer #2: Yes

Reviewer #3: Yes

**********

2. Has the statistical analysis been performed appropriately and rigorously?

Reviewer #1: Yes

Reviewer #2: I Don't Know

Reviewer #3: Yes

**********

3. Have the authors made all data underlying the findings in their manuscript fully available?

The PLOS Data policy

Reviewer #1: Yes

Reviewer #2: No

Reviewer #3: Yes

**********

4. Is the manuscript presented in an intelligible fashion and written in standard English?

Reviewer #1: Yes

Reviewer #2: Yes

Reviewer #3: Yes

**********

Reviewer #1: The manuscript is well written and highlights an important topic related to nutrient recovery and circular bioeconomy. The findings demonstrating that RO concentrate can be stored for six months without pasteurization are valuable. However, a few improvements are required:

Novelty clarification – The introduction should more clearly explain how this study advances existing research on RO/UF-based nutrient recovery.

Experimental limitations – Only one RAS source and batch were studied; please acknowledge this limitation and suggest future validation.

Statistical clarity – Figures should include statistical significance markers (p‐values, error bars clearly labelled).

Microbiological response – The significant viable cell increase after pasteurization needs deeper explanation.

Figures quality – Improve resolution and add clearer legends and units.

Reviewer #2: Your manuscript could be better if you compare your results with those main crops nutrient needs in Norway. Is interesting your foundings but it lacks of the support of the corps needs and more of what farmers need to improve their corps. This comparation will improve your work and give you more bases to have a more complete discussion obout your results. For now, your results are the preliminary part of what you really want to demonstrate with your research.

Reviewer #3: This is a valuable article in sustainable Aquacultur and Agriculture activities. Minor revisions are recommended.

I suggest to create a table for summerizing the chemical and microbial changes throughout the experimental period for both the Treated and the control Nutrient Concentrate.

**********

what does this mean?). If published, this will include your full peer review and any attached files.

If you choose “no”, your identity will remain anonymous but your review may still be made public.

**Do you want your identity to be public for this peer review?**  For information about this choice, including consent withdrawal, please see our Privacy Policy

Reviewer #1: No

Reviewer #2: No

Reviewer #3: No

**********

[NOTE: If reviewer comments were submitted as an attachment file, they will be attached to this email and accessible via the submission site. Please log into your account, locate the manuscript record, and check for the action link "View Attachments". If this link does not appear, there are no attachment files.]

To ensure your figures meet our technical requirements, please review our figure guidelines: https://journals.plos.org/plosone/s/figures

You may also use PLOS’s free figure tool, NAAS, to help you prepare publication quality figures: https://journals.plos.org/plosone/s/figures#loc-tools-for-figure-preparation.

NAAS will assess whether your figures meet our technical requirements by comparing each figure against our figure specifications.

Attachment Submitted filename: PONE-D-25-37522.docx

Attachment Submitted filename: PONE-D-25-37522 (reviewer comments).pdf

# Author comment: Author response to Decision Letter 1

**DOI:** 10.1371/journal.pone.0355602.r002

23 Dec 2025

Please see the file "response to reviewers" uploaded as a separate file as requested.

Attachment Submitted filename: Response to Reviewers.docx

# Peer review: Decision Letter 1

**DOI:** 10.1371/journal.pone.0355602.r003

**Authors:** Dave Mangindaan

24 Feb 2026

Dear Dr. Ulleberg,

Thank you for submitting your manuscript to PLOS ONE. After careful consideration, we feel that it has merit but does not fully meet PLOS ONE’s publication criteria as it currently stands. Therefore, we invite you to submit a revised version of the manuscript that addresses the points raised during the review process.

==============================  **From Academic Editor** **:**  **download**

In addition, please add Data Availability Statement, placed before the Acknowledgment

The data that support the findings of this study are openly available in OSF repository at https://osf.io/9bqpc/?view_only=b61606c3dd524ce3a9a3692b7dd7c81e

While it has been stated as Reference 15, but the addition of the Data Availability Statement (before the Acknowledgment) will not just highlight the source of the data (as asked by Reviewer #3), but also to comply with https://journals.plos.org/plosone/s/data-availability

Thank you.

==============================?>

Please submit your revised manuscript by Apr 10 2026 11:59PM. If you will need more time than this to complete your revisions, please reply to this message or contact the journal office at plosone@plos.org. When you're ready to submit your revision, log on to https://www.editorialmanager.com/pone/ and select the 'Submissions Needing Revision' folder to locate your manuscript file.

- A letter that responds to each point raised by the academic editor and reviewer(s). You should upload this letter as a separate file labeled 'Response to Reviewers'.
- A marked-up copy of your manuscript that highlights changes made to the original version. You should upload this as a separate file labeled 'Revised Manuscript with Track Changes'.
- An unmarked version of your revised paper without tracked changes. You should upload this as a separate file labeled 'Manuscript'.

If you would like to make changes to your financial disclosure, please include your updated statement in your cover letter. Guidelines for resubmitting your figure files are available below the reviewer comments at the end of this letter.

If applicable, we recommend that you deposit your laboratory protocols in protocols.io to enhance the reproducibility of your results. Protocols.io assigns your protocol its own identifier (DOI) so that it can be cited independently in the future. For instructions see: https://journals.plos.org/plosone/s/submission-guidelines#loc-laboratory-protocols. Additionally, PLOS ONE offers an option for publishing peer-reviewed Lab Protocol articles, which describe protocols hosted on protocols.io. Read more information on sharing protocols at https://plos.org/protocols?utm_medium=editorial-email&utm_source=authorletters&utm_campaign=protocols.

We look forward to receiving your revised manuscript.

Kind regards,

Dave Mangindaan

Academic Editor

PLOS One

Journal Requirements:

If the reviewer comments include a recommendation to cite specific previously published works, please review and evaluate these publications to determine whether they are relevant and should be cited. There is no requirement to cite these works unless the editor has indicated otherwise.

[Note: HTML markup is below. Please do not edit.]

Reviewers' comments:

Reviewer's Responses to Questions

**Comments to the Author**

Reviewer #1: All comments have been addressed

Reviewer #2: All comments have been addressed

**********

2. Is the manuscript technically sound, and do the data support the conclusions?

Reviewer #1: Yes

Reviewer #2: Yes

**********

3. Has the statistical analysis been performed appropriately and rigorously?

Reviewer #1: Yes

Reviewer #2: I Don't Know

**********

4. Have the authors made all data underlying the findings in their manuscript fully available?

The PLOS Data policy

Reviewer #1: Yes

Reviewer #2: Yes

**********

5. Is the manuscript presented in an intelligible fashion and written in standard English?

Reviewer #1: Yes

Reviewer #2: Yes

**********

Reviewer #1: The manuscript presents valuable findings with practical implications for aquaculture and sustainable agriculture.

Reviewer #2: The arguments in their results are very weak and require further review of the literature. Most of your assumptions lack of technical and literature support in each area of their results. I recommend conducting a more in-depth literary review to support your arguments and the evidence you've found in your results.

**********

what does this mean?). If published, this will include your full peer review and any attached files.

If you choose “no”, your identity will remain anonymous but your review may still be made public.

**Do you want your identity to be public for this peer review?**  For information about this choice, including consent withdrawal, please see our Privacy Policy

Reviewer #1: No

Reviewer #2: **Yes:**  Gerardo Rodríguez Quiroz

**********

[NOTE: If reviewer comments were submitted as an attachment file, they will be attached to this email and accessible via the submission site. Please log into your account, locate the manuscript record, and check for the action link "View Attachments". If this link does not appear, there are no attachment files.]

To ensure your figures meet our technical requirements, please review our figure guidelines: https://journals.plos.org/plosone/s/figures

You may also use PLOS’s free figure tool, NAAS, to help you prepare publication quality figures: https://journals.plos.org/plosone/s/figures#loc-tools-for-figure-preparation.

NAAS will assess whether your figures meet our technical requirements by comparing each figure against our figure specifications.

Attachment Submitted filename: minor revision.docx

# Author comment: Author response to Decision Letter 2

**DOI:** 10.1371/journal.pone.0355602.r004

19 Mar 2026

The response to comments from each reviewer and the editor are found in the document "Response to reviewers".

Attachment Submitted filename: Response_to_Reviewers_auresp_2.docx

# Peer review: Decision Letter 2

**DOI:** 10.1371/journal.pone.0355602.r005

**Authors:** Dave Mangindaan

30 Mar 2026

Dear Dr. Ulleberg,

Thank you for submitting your manuscript to PLOS ONE. After careful consideration, we feel that it has merit but does not fully meet PLOS ONE’s publication criteria as it currently stands. Therefore, we invite you to submit a revised version of the manuscript that addresses the points raised during the review process.

**From Academic Editor:**

Please submit your revised manuscript by May 14 2026 11:59PM. If you will need more time than this to complete your revisions, please reply to this message or contact the journal office at plosone@plos.org. When you're ready to submit your revision, log on to https://www.editorialmanager.com/pone/ and select the 'Submissions Needing Revision' folder to locate your manuscript file.

- A letter that responds to each point raised by the academic editor and reviewer(s). You should upload this letter as a separate file labeled 'Response to Reviewers'.
- A marked-up copy of your manuscript that highlights changes made to the original version. You should upload this as a separate file labeled 'Revised Manuscript with Track Changes'.
- An unmarked version of your revised paper without tracked changes. You should upload this as a separate file labeled 'Manuscript'.

If you would like to make changes to your financial disclosure, please include your updated statement in your cover letter. Guidelines for resubmitting your figure files are available below the reviewer comments at the end of this letter.

If applicable, we recommend that you deposit your laboratory protocols in protocols.io to enhance the reproducibility of your results. Protocols.io assigns your protocol its own identifier (DOI) so that it can be cited independently in the future. For instructions see: https://journals.plos.org/plosone/s/submission-guidelines#loc-laboratory-protocols. Additionally, PLOS ONE offers an option for publishing peer-reviewed Lab Protocol articles, which describe protocols hosted on protocols.io. Read more information on sharing protocols at https://plos.org/protocols?utm_medium=editorial-email&utm_source=authorletters&utm_campaign=protocols.

As the corresponding author, your ORCID iD is verified in the submission system and will appear in the published article. PLOS supports the use of ORCID, and we encourage all coauthors to register for an ORCID iD and use it as well. Please encourage your coauthors to verify their ORCID iD within the submission system before final acceptance, as unverified ORCID iDs will not appear in the published article. *Only*  the individual author can complete the verification step; PLOS staff *cannot*  verify ORCID iDs on behalf of authors.

We look forward to receiving your revised manuscript.

Kind regards,

Assoc. Prof. Dave Mangindaan, PhD, MRSC, AMIChemE.

Academic Editor

PLOS One

Journal Requirements:

If the reviewer comments include a recommendation to cite specific previously published works, please review and evaluate these publications to determine whether they are relevant and should be cited. There is no requirement to cite these works unless the editor has indicated otherwise.

[Note: HTML markup is below. Please do not edit.]

Reviewers' comments:

Reviewer's Responses to Questions

**Comments to the Author**

Reviewer #1: All comments have been addressed

Reviewer #2: All comments have been addressed

**********

2. Is the manuscript technically sound, and do the data support the conclusions?

Reviewer #1: Yes

Reviewer #2: Yes

**********

3. Has the statistical analysis been performed appropriately and rigorously?

Reviewer #1: Yes

Reviewer #2: Yes

**********

4. Have the authors made all data underlying the findings in their manuscript fully available?

The PLOS Data policy

Reviewer #1: Yes

Reviewer #2: Yes

**********

5. Is the manuscript presented in an intelligible fashion and written in standard English?

Reviewer #1: Yes

Reviewer #2: Yes

**********

Reviewer #1: Major concerns:

• Experimental design is limited (single source, single batch; microbiology n=1), reducing reliability.

• Statistical analysis is inadequate; time-series data require more robust methods.

• Claims of “microbial stability” are somewhat overstated given observed fluctuations.

• Mechanisms behind nutrient loss after pasteurization are speculative and need validation.

• No agronomic validation (e.g., plant trials) to support fertilizer applicability.

Minor concerns:

• Improve clarity, reduce repetition, and refine language.

• Correct duplicated figure captions and improve figure presentation.

• Ensure consistency in units and terminology (avoid labeling as purely “organic fertilizer”).

Recommendation:

Major Revision – The study is promising but requires methodological strengthening and clearer interpretation before publication

Reviewer #2: (No Response)

**********

what does this mean?). If published, this will include your full peer review and any attached files.

If you choose “no”, your identity will remain anonymous but your review may still be made public.

**Do you want your identity to be public for this peer review?**  For information about this choice, including consent withdrawal, please see our Privacy Policy

Reviewer #1: No

Reviewer #2: **Yes:**  Gerardo Rodriguez Quiroz

**********

[NOTE: If reviewer comments were submitted as an attachment file, they will be attached to this email and accessible via the submission site. Please log into your account, locate the manuscript record, and check for the action link "View Attachments". If this link does not appear, there are no attachment files.]

To ensure your figures meet our technical requirements, please review our figure guidelines: https://journals.plos.org/plosone/s/figures

You may also use PLOS’s free figure tool, NAAS, to help you prepare publication quality figures: https://journals.plos.org/plosone/s/figures#loc-tools-for-figure-preparation.

NAAS will assess whether your figures meet our technical requirements by comparing each figure against our figure specifications.

# Author comment: Author response to Decision Letter 3

**DOI:** 10.1371/journal.pone.0355602.r006

23 Apr 2026

Response to Reviewers

Dear Editor, please find below our responses to the reviewers’ comments. The accompanying file, Revised Manuscript with Track Changes, highlights all modifications made in response to each comment.

From Academic Editor:

Please download the attachment from Reviewer 1. There are also still many issues to clarify per Reviewer 1's suggestions. In addition, the data shown in the manuscript are two figures (with one with 5 panels, and the second is just qualitative/visual image). I agree with Reviewer 1 who requires new data (and discussions), with the objective to have a solid manuscript.

Dear Editor,

Thank you for your feedback. We have addressed the comments from Reviewer 1 below.

With regard to the data presented in the Results and Discussion section, we would like to respectfully clarify that the manuscript includes more quantitative data than what is implied in your comment.

Specifically, Table 1 presents the initial composition of the nutrient concentrate, Figure 2 presents the change of chemical parameters during storage, and Figure 3 presents the change of microbiological parameters during storage. Of these, you only include figure 2 in your comment. In addition, the complete chemical composition and additional microbiological parameters are provided in the Supplementary Materials available at https://osf.io/9bqpc/overview?view_only=b61606c3dd524ce3a9a3692b7dd7c81e. As you point out figure 1 is only a visual image, which we have included in the article to provide context for the reader.

We therefore argue that the manuscript contains sufficient data for publication.

Reviewers' comments:

Reviewer's Responses to Questions

Comments to the Author

1. If the authors have adequately addressed your comments raised in a previous round of review and you feel that this manuscript is now acceptable for publication, you may indicate that here to bypass the “Comments to the Author” section, enter your conflict of interest statement in the “Confidential to Editor” section, and submit your "Accept" recommendation.

Reviewer #1: All comments have been addressed

Reviewer #2: All comments have been addressed

2. Is the manuscript technically sound, and do the data support the conclusions?

The manuscript must describe a technically sound piece of scientific research with data that supports the conclusions. Experiments must have been conducted rigorously, with appropriate controls, replication, and sample sizes. The conclusions must be drawn appropriately based on the data presented.

Reviewer #1: Yes

Reviewer #2: Yes

3. Has the statistical analysis been performed appropriately and rigorously?

Reviewer #1: Yes

Reviewer #2: Yes

4. Have the authors made all data underlying the findings in their manuscript fully available?

Reviewer #1: Yes

Reviewer #2: Yes

5. Is the manuscript presented in an intelligible fashion and written in standard English?

PLOS ONE does not copyedit accepted manuscripts, so the language in submitted articles must be clear, correct, and unambiguous. Any typographical or grammatical errors should be corrected at revision, so please note any specific errors here.

Reviewer #1: Yes

Reviewer #2: Yes

6. Review Comments to the Author

Please use the space provided to explain your answers to the questions above. You may also include additional comments for the author, including concerns about dual publication, research ethics, or publication ethics. (Please upload your review as an attachment if it exceeds 20,000 characters)

Reviewer #1: Major concerns:

• Experimental design is limited (single source, single batch; microbiology n=1), reducing reliability.

The inorganic concentrate used in this study was produced in a unique reverse osmosis pilot system permanently connected to an experimental RAS facility owned by NIBIO Landvik. Including multiple sources of RAS effluent water was therefore not practically feasible. In the case of data derived by flow cytometry, single samples were analysed as described in Prest et al (2013), https://doi.org/10.1016/j.watres.2013.07.051, which documents the extremely low measurement variability when using this method mainly due to the statistical replication inherent in a single sample containing millions of individually counted cells. While we believe this study is a valuable contribution to the literature in its current form, we have acknowledged the limitations highlighted in this review in our conclusion, which includes the following statement:

“As this study relied on a single source of RAS wastewater, future studies should include multiple sources of RAS wastewater and different batches of RO concentrate to further validate the findings.”

• Statistical analysis is inadequate; time-series data require more robust methods.

As stated in the previous comment, we acknowledge the limitations of the study in the conclusion with the statement “As this study relied on a single source of RAS wastewater, future studies should include multiple sources of RAS wastewater and different batches of RO concentrate to further validate the findings.” Otherwise, appropriate statistical methods were used to analyse the results, given the experimental design. We would also like to point out that both reviewers have answered “yes” to the question of whether statistical analysis been performed appropriately and rigorously above.

• Claims of “microbial stability” are somewhat overstated given observed fluctuations.

We argue that the system is microbiologically stable because we do not observe exponential growth or large fluctuations over time as one would expect in a system dominated by microorganisms if conditions favoured growth. The observed fluctuations are limited to the samples that were pasteurised, where an initial reduction in the viable cell load and intact bacterial cells quickly recovered to a level which remained stable during the storage period independent of treatment.

• Mechanisms behind nutrient loss after pasteurization are speculative and need validation.

We agree that the mechanisms behind nutrient loss after pasteurization need validation in future studies that are designed to investigate these observations. However, the scope of this study was to investigate the storage stability of an inorganic concentrate produced from RAS effluent water with and without pasteurization. It is, to our knowledge, the first time this specific concept has been studied, and is an important contribution towards the development of viable alternatives to conventional mineral fertilizers.

• No agronomic validation (e.g., plant trials) to support fertilizer applicability.

This study was designed to investigate the storage stability of an inorganic concentrate produced from RAS effluent water with and without pasteurization. Agronomic validation of the fertilizer applicability will be evaluated in future plant trials, as we have recommended in the conclusion section of the manuscript.

Minor concerns:

• Improve clarity, reduce repetition, and refine language.

We have edited the manuscript to improve clarity and reduce repetition. We would also like to point out that both reviewers have answered “yes” to the question of whether the manuscript is presented in an intelligible fashion above.

• Correct duplicated figure captions and improve figure presentation.

We have ensured consistent figure captions and references throughout the text.

• Ensure consistency in units and terminology (avoid labeling as purely “organic fertilizer”).

We have reviewed and altered the labelling, units and terminology throughout the manuscript.

Attachment Submitted filename: Response_to_Reviewers_auresp_3.docx

# Peer review: Decision Letter 3

**DOI:** 10.1371/journal.pone.0355602.r007

**Authors:** Dave Mangindaan

26 May 2026

Dear Dr. Ulleberg,

Thank you for submitting your manuscript to PLOS ONE. After careful consideration, we feel that it has merit but does not fully meet PLOS ONE’s publication criteria as it currently stands. Therefore, we invite you to submit a revised version of the manuscript that addresses the points raised during the review process.

==============================

Please submit your revised manuscript by Jul 10 2026 11:59PM. If you will need more time than this to complete your revisions, please reply to this message or contact the journal office at plosone@plos.org. When you're ready to submit your revision, log on to https://www.editorialmanager.com/pone/ and select the 'Submissions Needing Revision' folder to locate your manuscript file.

- A letter that responds to each point raised by the academic editor and reviewer(s). You should upload this letter as a separate file labeled 'Response to Reviewers'.
- A marked-up copy of your manuscript that highlights changes made to the original version. You should upload this as a separate file labeled 'Revised Manuscript with Track Changes'.
- An unmarked version of your revised paper without tracked changes. You should upload this as a separate file labeled 'Manuscript'.

If you would like to make changes to your financial disclosure, please include your updated statement in your cover letter. Guidelines for resubmitting your figure files are available below the reviewer comments at the end of this letter.

If applicable, we recommend that you deposit your laboratory protocols in protocols.io to enhance the reproducibility of your results. Protocols.io assigns your protocol its own identifier (DOI) so that it can be cited independently in the future. For instructions see: https://journals.plos.org/plosone/s/submission-guidelines#loc-laboratory-protocols. Additionally, PLOS ONE offers an option for publishing peer-reviewed Lab Protocol articles, which describe protocols hosted on protocols.io. Read more information on sharing protocols at https://plos.org/protocols?utm_medium=editorial-email&utm_source=authorletters&utm_campaign=protocols.

As the corresponding author, your ORCID iD is verified in the submission system and will appear in the published article. PLOS supports the use of ORCID, and we encourage all coauthors to register for an ORCID iD and use it as well. Please encourage your coauthors to verify their ORCID iD within the submission system before final acceptance, as unverified ORCID iDs will not appear in the published article. *Only*  the individual author can complete the verification step; PLOS staff *cannot*  verify ORCID iDs on behalf of authors.

We look forward to receiving your revised manuscript.

Kind regards,

Dave Mangindaan

Academic Editor

PLOS One

**Journal Requirements:**

1. If the reviewer comments include a recommendation to cite specific previously published works, please review and evaluate these publications to determine whether they are relevant and should be cited. There is no requirement to cite these works unless the editor has indicated otherwise.

[Note: HTML markup is below. Please do not edit.]

Reviewers' comments:

Reviewer's Responses to Questions

**Comments to the Author**

Reviewer #1: All comments have been addressed

Reviewer #2: (No Response)

Reviewer #4: (No Response)

Reviewer #5: (No Response)

Reviewer #6: (No Response)

**********

2. Is the manuscript technically sound, and do the data support the conclusions?

Reviewer #1: Yes

Reviewer #2: Partly

Reviewer #4: (No Response)

Reviewer #5: Partly

Reviewer #6: Yes

**********

3. Has the statistical analysis been performed appropriately and rigorously?

Reviewer #1: Yes

Reviewer #2: Yes

Reviewer #4: (No Response)

Reviewer #5: No

Reviewer #6: Yes

**********

4. Have the authors made all data underlying the findings in their manuscript fully available?

The PLOS Data policy

Reviewer #1: Yes

Reviewer #2: Yes

Reviewer #4: (No Response)

Reviewer #5: Yes

Reviewer #6: Yes

**********

5. Is the manuscript presented in an intelligible fashion and written in standard English?

Reviewer #1: Yes

Reviewer #2: Yes

Reviewer #4: (No Response)

Reviewer #5: Yes

Reviewer #6: Yes

**********

Reviewer #1: all comments given are done appropriately. this is important paper in advanced aquaculture field where RAS technique is one of that.

Reviewer #2: (No Response)

Reviewer #4: This work recovered plant nutrients from salmon RAS effluent using a RO system and studied the storage stability of the resulting RO concentrate over six months. It is concluded that RAS NC is stable when stored at room temperature without light for six months, regardless of pasteurization. Some more comments are:

1. Flocculation, UF and RO are used to treat the RAS effluent. More discussion and data on the system should be provided. For example, what are the size, rejection, recovery of the UF and RO units? What are the requirements for plant nutrients in terms of composition and concentration? How is RO concentrate compared to the requirements?

2. Why is pasteurization (heat treatment) carried out at 80 oC for 10 minutes? Is this an optimum condition?

3. Visual assessment of physical characteristics revealed no change in precipitation. Why is precipitation highlighted as the reason for reduced Fe concentration?

4. It is hypothesized that the observed increase in the solution’s pH has resulted in

precipitation of iron and other dissolved ions. This claim needs to be supported with calculation. The pH is increased from 6.97 to 7.37. How much precipitation can happen with this change of pH?

5. With heat treatment, why is the pH increased from 6.97 to 7.37?

6. It is said that EC indicates temporal fluctuations in the degree of mineral precipitation during the storage period. Why are there temporal fluctuations?

7. The viable cell load increased slightly in the unpasteurized control towards the end of the storage period, which may indicate the onset of spoilage. Do this mean the pasteurized sample have longer storage stability?

8. UF is effective in removing microorganisms. Can you use UF to pasteurize the sample, in steady of 80 oC for 10 minutes?

Reviewer #5: The author needs to address these to improve their study:

1. There is substantial research on using RO after effluent treatment, with a strong focus on the resulting concentrate. Pasteurization has also been used to improve the stability of the effluent. So, what is actually the novelty of this work compared to those reported works? What is the main scientific contribution of recovering liquid fertilizer from RAS effluent using reverse osmosis?

2. I also did not find why RO is selected over other nutrient concentration technologies, such as nanofiltration, forward osmosis, membrane distillation, evaporation, or adsorption. There is no systematic approach to selecting RO in their work.

3. What about RAS composition before RO treatment? I found only RAS NC composition and characteristics. They need to compare the composition before and after RO treatment.

4. Figure 1 shows physical characteristics. I was thinking it was something like density and other physical properties. But it is basically only the image of the RAS. This is not physical characteristics. It is just physical appearance. So better to use another caption to avoid misleading about something else.

5. Does the recovered RO concentrate meet basic requirements for use as a liquid fertilizer? I mean, let’s say by comparing the recovered fertilizer with commercial liquid fertilizers?

6. Under the tested dark-storage conditions, pasteurization did not significantly affect the measured microbial load, intact bacterial cell concentration, or bacterial diversity of the RO-concentrated RAS liquid fertilizer. But it is not sufficient on its own to support a broad practical or commercial claim that pasteurization is unnecessary or obsolete as they concluded. They should test specifically for safety-relevant microorganisms, not only total viable cells to provide strong claim on

7. Could the process support circular economy principles in aquaculture-agriculture integration?

Reviewer #6: 1. Please add a flowchart to show more clearly the experimental process.

2. The conclusion that the concentrate is “microbiologically stable” may be somewhat overstated considering the observed increase in viable cell load and reduction in bacterial diversity toward the end of the storage period in the unpasteurized samples. These trends may indicate the early onset of spoilage, and the discussion should acknowledge this limitation more cautiously.

3. Although the study demonstrates storage stability, the practical fertilizer applicability of the recovered concentrate is not experimentally validated. No plant growth trials, nutrient uptake studies, or agronomic performance assessments were conducted to confirm the effectiveness of the product as an alternative fertilizer source. Including preliminary cultivation experiments would substantially enhance the practical significance of the manuscript.

**********

what does this mean?). If published, this will include your full peer review and any attached files.

If you choose “no”, your identity will remain anonymous but your review may still be made public.

**Do you want your identity to be public for this peer review?**  For information about this choice, including consent withdrawal, please see our Privacy Policy

Reviewer #1: No

Reviewer #2: **Yes:**  Gerardo Rodriguez Quiroz

Reviewer #4: No

Reviewer #5: No

Reviewer #6: No

**********

[NOTE: If reviewer comments were submitted as an attachment file, they will be attached to this email and accessible via the submission site. Please log into your account, locate the manuscript record, and check for the action link "View Attachments". If this link does not appear, there are no attachment files.]

To ensure your figures meet our technical requirements, please review our figure guidelines: https://journals.plos.org/plosone/s/figures

You may also use PLOS’s free figure tool, NAAS, to help you prepare publication quality figures: https://journals.plos.org/plosone/s/figures#loc-tools-for-figure-preparation.

NAAS will assess whether your figures meet our technical requirements by comparing each figure against our figure specifications.

# Author comment: Author response to Decision Letter 4

**DOI:** 10.1371/journal.pone.0355602.r008

7 Jul 2026

Please see the file labeled 'Response to Reviewers' for a letter that responds to each point raised by the academic editor and reviewer(s), as instructed in the decision letter.

Attachment Submitted filename: Response_to_Reviewers_auresp_4.docx

# Peer review: Decision Letter 4

**DOI:** 10.1371/journal.pone.0355602.r009

**Authors:** Sovik Das

24 Jul 2026

Liquid fertilizer recovered from the effluent water of recirculating aquaculture system by reverse osmosis exhibits storage stability independent of pasteurization

PONE-D-25-37522R4

Dear Dr. Ulleberg,

We’re pleased to inform you that your manuscript has been judged scientifically suitable for publication and will be formally accepted for publication once it meets all outstanding technical requirements.

Within one week, you’ll receive an e-mail detailing the required amendments. When these have been addressed, you’ll receive a formal acceptance letter and your manuscript will be scheduled for publication.

An invoice will be generated when your article is formally accepted. Please note, if your institution has a publishing partnership with PLOS and your article meets the relevant criteria, all or part of your publication costs will be covered. Please make sure your user information is up-to-date by logging into Editorial Manager at Editorial Manager® and clicking the ‘Update My Information' link at the top of the page. For questions related to billing, please contact billing support.

If your institution or institutions have a press office, please notify them about your upcoming paper to help maximize its impact. If they’ll be preparing press materials, please inform our press team as soon as possible -- no later than 48 hours after receiving the formal acceptance. Your manuscript will remain under strict press embargo until 2 pm Eastern Time on the date of publication. For more information, please contact onepress@plos.org.

Kind regards,

Sovik Das

Academic Editor

PLOS One

Additional Editor Comments (optional):

Reviewers' comments:

Reviewer's Responses to Questions

**Comments to the Author**

Reviewer #1: All comments have been addressed

Reviewer #2: All comments have been addressed

Reviewer #6: All comments have been addressed

**********

2. Is the manuscript technically sound, and do the data support the conclusions?

Reviewer #1: Yes

Reviewer #2: Yes

Reviewer #6: Yes

**********

3. Has the statistical analysis been performed appropriately and rigorously?

Reviewer #1: Yes

Reviewer #2: Yes

Reviewer #6: Yes

**********

4. Have the authors made all data underlying the findings in their manuscript fully available?

The PLOS Data policy

Reviewer #1: Yes

Reviewer #2: Yes

Reviewer #6: Yes

**********

5. Is the manuscript presented in an intelligible fashion and written in standard English?

Reviewer #1: Yes

Reviewer #2: Yes

Reviewer #6: Yes

**********

Reviewer #1: author have given all answered. Accept the paper. its focused on advanced aquaculture system and this system is sustainable.

Reviewer #2: All the recommendations had been addressed. I hope your paper could contribuite to the aquaculture in the further future

Reviewer #6: Authors have added necessary explanations and figures, and they have responded appropriately to all my comments.

**********

what does this mean?). If published, this will include your full peer review and any attached files.

If you choose “no”, your identity will remain anonymous but your review may still be made public.

**Do you want your identity to be public for this peer review?**  For information about this choice, including consent withdrawal, please see our Privacy Policy

Reviewer #1: No

Reviewer #2: No

Reviewer #6: No

**********

# Editor report: Acceptance letter

**DOI:** 10.1371/journal.pone.0355602.r010

**Authors:** Sovik Das

PONE-D-25-37522R4

PLOS One

Dear Dr. Ulleberg,

I'm pleased to inform you that your manuscript has been deemed suitable for publication in PLOS One. Congratulations! Your manuscript is now being handed over to our production team.

At this stage, our production department will prepare your paper for publication. This includes ensuring the following:

* All references, tables, and figures are properly cited

* All relevant supporting information is included in the manuscript submission,

* There are no issues that prevent the paper from being properly typeset

You will receive further instructions from the production team, including instructions on how to review your proof when it is ready. Please keep in mind that we are working through a large volume of accepted articles, so please give us a few days to review your paper and let you know the next and final steps.

Lastly, if your institution or institutions have a press office, please let them know about your upcoming paper now to help maximize its impact. If they'll be preparing press materials, please inform our press team within the next 48 hours. Your manuscript will remain under strict press embargo until 2 pm Eastern Time on the date of publication. For more information, please contact onepress@plos.org.

You will receive an invoice from PLOS for your publication fee after your manuscript has reached the completed accept phase. If you receive an email requesting payment before acceptance or for any other service, this may be a phishing scheme. Learn how to identify phishing emails and protect your accounts at https://explore.plos.org/phishing.

If we can help with anything else, please email us at customercare@plos.org.

Thank you for submitting your work to PLOS One and supporting open access.

Kind regards,

PLOS ONE Editorial Office Staff

on behalf of

Prof Sovik Das

Academic Editor

PLOS One
